# *Bacillus altitudinis* AD13−4 Enhances Saline–Alkali Stress Tolerance of Alfalfa and Affects Composition of Rhizosphere Soil Microbial Community

**DOI:** 10.3390/ijms25115785

**Published:** 2024-05-26

**Authors:** Muneer Ahmed Khoso, Mingyu Wang, Zhenzhen Zhou, Yongxue Huang, Shenglin Li, Yiming Zhang, Guangtao Qian, Song Nam Ko, Qiuying Pang, Changli Liu, Lixin Li

**Affiliations:** 1 Key Laboratory of Saline-Alkali Vegetation Ecology Restoration, Ministry of Education, College of Life Sciences, Northeast Forestry University, Harbin 150040, China; mak6814@yahoo.com (M.A.K.); wmy19970825@163.com (M.W.); zzz_1204@126.com (Z.Z.); hyxcsbh@163.com (Y.H.); 13664522543@163.com (S.L.); syzdxk@163.com (Y.Z.); qgtwu20@163.com (G.Q.); ksn198023@163.com (S.N.K.); qiuying@nefu.edu.cn (Q.P.); 2 College of Life Sciences and Agriculture and Forestry, Qiqihar University, Qiqihar 161006, China

**Keywords:** alkaline–sodic stress, PGPR, rhizosphere microbiota, plant–PGPR interaction, signal transduction, secondary metabolism

## Abstract

Saline and alkaline stresses limit plant growth and reduce crop yield. Soil salinization and alkalization seriously threaten the sustainable development of agriculture and the virtuous cycle of ecology. Biofertilizers made from plant growth−promoting rhizobacteria (PGPR) not only enhance plant growth and stress tolerance, but also are environmentally friendly and cost-effective. There have been many studies on the mechanisms underlying PGPRs enhancing plant salt resistance. However, there is limited knowledge about the interaction between PGPR and plants under alkaline–sodic stress. To clarify the mechanisms underlying PGPR’s improvement of plants’ tolerance to alkaline–sodic stress, we screened PGPR from the rhizosphere microorganisms of local plants growing in alkaline–sodic land and selected an efficient strain, *Bacillus altitudinis* AD13−4, as the research object. Our results indicate that the strain AD13−4 can produce various growth-promoting substances to regulate plant endogenous hormone levels, cell division and differentiation, photosynthesis, antioxidant capacity, etc. Transcriptome analysis revealed that the strain AD13−4 significantly affected metabolism and secondary metabolism, signal transduction, photosynthesis, redox processes, and plant–pathogen interactions. Under alkaline–sodic conditions, inoculation of the strain AD13−4 significantly improved plant biomass and the contents of metabolites (e.g., soluble proteins and sugars) as well as secondary metabolites (e.g., phenols, flavonoids, and terpenoids). The 16S rRNA gene sequencing results indicated that the strain AD13−4 significantly affected the abundance and composition of the rhizospheric microbiota and improved soil activities and physiochemical properties. Our study provides theoretical support for the optimization of saline–alkali-tolerant PGPR and valuable information for elucidating the mechanism of plant alkaline–sodic tolerance.

## 1. Introduction

Soil salinity and alkalinity represent one of the major global environmental issues that threaten food security and agricultural sustainability. The total area of saline–alkali land in China is about 9.91 × 10^7^ ha [1]. In northeast China, the saline–alkali land mainly contains high concentration of carbonates, including Na_2_CO_3_ and NaHCO_3_; therefore, it is also known as Soda saline–alkali land [1,2,3]. The presence of sodium ions in the soil induces stress, which significantly hampers plant growth [4]. On the other hand, the saline–alkali stress caused by carbonates has more serious impacts on plants than salt stress [5]. Because, in addition to osmotic stress, ion toxicity, and oxidative stress, which are usually caused by salt stress [6,7], alkali stress also causes nutritional deficiency and high pH stress [1,8,9]. The nutrient deficiency mainly refers to the unavailability of nutrients such as phosphorus and iron ions due to the precipitation in alkaline soil [10,11]. Compound saline and alkali stresses can lead to more severe oxidative stress, disruption of ion homeostasis, and metabolic disorders [12,13,14], leading to damage to cell structure and activities, thereby inhibiting plant growth and development. To re–establish osmotic and ion homeostasis to adapt to adverse environments, plant cells rapidly accumulate inorganic ions [15,16] and small organic molecules such as betaine, proline, polyamines, polyols, and sugars [17,18]. For instance, the trehalose biosynthesis process in quinoa leaves is significantly affected under saline–sodic stress [19]. In addition to highly accumulated lipids and amino acids, the energy metabolism and reactive oxygen species (ROS)-scavenging machineries in rice leaves are significantly enhanced under severe saline–sodic stress, such as tricarboxylic acid (TCA) cycle and glutathione metabolism [1]. The accumulation of proline and its derivatives in rice leaves facilitates osmotic balance control, thereby improving plant saline–alkali tolerance [1,20].

Considering the potential threats of saline–alkali stress on ecosystems and food security, significant efforts are needed to improve saline–alkali land. Plant growth–promoting rhizobacteria (PGPRs) play a crucial role in plant growth [21]. For instance, inoculation of *Sinorhizobium meliloti* GL1 and *Enterobacter ludwigii* MJM–11 improves the yield, nodulation, and quality of alfalfa in a saline–alkali environment [22,23]. *Enterobacter asburiae* strain D2 has the ability to produce 1–aminocyclop ropane–1–carboxylate (ACC) deaminase, indole−3−acetic acid (IAA), and siderophore, as well as to solubilize phosphate. It may alleviate the impacts of saline–alkali stress on rice [24]. *Bacillus subtilis* has been found to enhance crop saline–alkali stress tolerance by preventing excessive sodium accumulation and enhancing nutrient absorption. Application of the bacteria enhances the activities of peroxidase and catalase in leaves, thereby protecting plants from salt stress–induced damage [25]. A combination of *Enterobacter* sp. Z1 and *Klebsiella* sp. Z2 can significantly improve soybean growth and nitrogen fixation by producing flavonoids, IAA, salicylic acid (SA), and taurine (51). *Enterobacter aerogenes* (LJL–5) and *Pseudomonas aeruginosa* (LJL–13) synergistically increased the biomass of alfalfa plants and improved phosphorus content and antioxidase activities (superoxide dismutase, peroxidase, and catalase) in saline–alkali conditions [26]. A research has shown that the strains *Stutzerimonas stutzeri* A38 and *Bacillus pumilus* A49 have the ability to enhance root size in *Medicago sativa* and *Medicago polymorpha* plants when exposed to osmotic stress [4]. In addition, PGPRs can also protect plants from heavy metal pollution by regulating the levels of plant endogenous SA, abscisic acid (ABA), and jasmonic acid (JA) [27,28]. *Bacillus* sp. ZC3–2–1 can improve the phytoremediation efficiency of Cd–Zn–contaminated soil and maintain ion homeostasis by promoting the phytoextraction and immobilization of the metal ions [29,30]. Thus, PGPRs are widely used as biofertilizers to improve crop biomass and yields, and as soil amendments to improve land availability [31,32,33]. Due to its low carbon, environmental friendliness, and cost-effectiveness, PGPR has obtained increasing public acceptance [33,34].

The diversity of microorganisms associated with plant roots with approximately tens of thousands of species, known as the plant’s second genome, plays vital roles in plant development, health, and environmental adaptation in their natural habitats [35,36]. For example, the presence of *Enterobacterium* and *Pseudomonas* in soil indicates that exotic species highly rely on rhizosphere microorganisms to fulfill their nutritional needs [37]. The important role of co–evolution and interaction between plants and microorganisms suggest that plants can shape their rhizosphere microbial communities, which proves that different plant species have specific microbial communities when grown in the same soil [38,39]. This complex relationship goes beyond the rhizosphere and involves a diverse range of interactions that have a significant influence on plant vitality and the dynamics of the ecosystem [23,40,41]. Furthermore, recent progress in high–throughput sequencing and omics technologies has given a deeper understanding of the functions and metabolic processes of rhizosphic microbiota [42], revealing the complex involvement of rhizosphic microorganisms in nutrient cycling, the formation of soil structure, and the regulation of plant signaling pathways [42,43]. The acquisition of this knowledge has facilitated the development of novel approaches to exploiting plant–related advantageous characteristics of rhizosphic microorganisms [35]. Furthermore, the recently developed field of microbiome engineering has shown the potential to intentionally alter the rhizosphere microbial populations to enhance plant production and adaptability to environmental changes [44,45].

Various studies have been conducted to investigate the potential role of PGPR in alleviating the impacts of environmental stresses on plants while increasing biomass and yield (Supplementary Table S1). However, there is limited knowledge about the interactions between PGPR and plants under alkaline–sodic stress caused by carbonates. To clarify the mechanisms underlying PGPRs which improve the tolerance of plants to alkaline–sodic stress and the plants response to the PGPR, we isolated PGPRs from the rhizosphere microorganisms of local plants grown in Soda saline–alkali land; selected an effective strain, *Bacillus altitudinis* AD13−4; and explored the mechanisms underlying its growth–promoting function. Via biochemistry, molecular biology, and transcriptomics analysis, the strain AD13−4 was clarified to improve plant adaptation to alkaline–sodic environments by regulating plant metabolism, signal transduction, and plant–pathogen interaction, as well as by affecting the abundance and composition of the rhizosphere microbial community. Our study provides theoretical support for the optimization of saline–alkali–tolerant PGPR and valuable information for elucidating the alkaline–sodic tolerance mechanism of plants.

## 2. Results

### 2.1. Bacillus Altitudinis AD13−4 Promoted Plant Growth and Development under Saline–Alkali Stress

**Figure 1:**
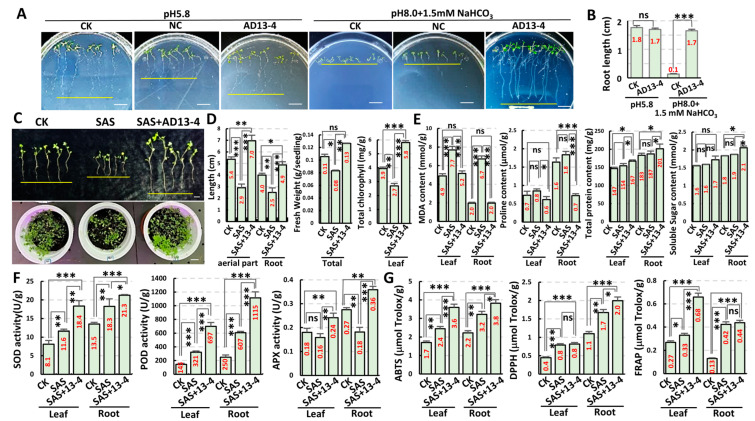
Strain AD13−4 significantly promoted plant growth. (**A**) Ten−day−old *Arabidopsis* seedlings grown on 1/2MS medium (pH 5.8) or 1/2MS medium (pH 8.0) containing 1 mM NaHCO_3_ (pH 8.0 + 1 mM NaHCO_3_) with or without inoculation of AD13−4. The yellow lines indicate the root tips. Bars = 1 cm. (**B**) Statistics of root length of seedlings shown in (**A**). (**C**) One−month−old alfalfa plants. SAS, saline–alkali stress. Bars = 5 cm. (**D**) Statistics of plant height, root length, fresh weight, and chlorophyll content in (**C**). (**E**) Statistics of the contents of malondialdehyde (MDA), proline, total protein and soluble sugars in (**C**). (**F**) Statistics of activities of antioxidant enzymes in (**C**). APX, ascorbate peroxidase; SOD, superoxide dismutase; POD, peroxidase. (**G**) Statistics of total ROS scavenging capacity. ABST, 2,2′−azino−bis (3−ethylbenzothiazoline−6−sulfonicacid); DPPH, 2,2−diphenyl−1−picrylhydrazyl; FRAP, ferric-reducing ability of plasma. For the statistics, three biological replicates per sample were used. *, *p* < 0.05; **, *p* < 0.01; ***, *p* < 0.001; ns, no significance. Student’s *t*−test. The numbers shown inside each column indicate the statistics value. CK, blank; NC, negative control.

To illustrate the growth–promoting mechanisms of PGPR under alkaline–sodic stress, we screened PGPR using carbonate medium (1/2MS, pH 8.0, 1.5 mM NaHCO_3_) to simulate an alkaline–sodic condition. Using *Arabidopsis* as the plant material, we obtained over 400 PGPR from the rhizosphere microorganisms of local plants growing in Soda saline–alkali land (Anda, Heilongjiang Province, China) and selected an effective strain, AD13−4, for mechanism investigation. As shown in Figure 1A, under normal conditions (pH 5.8), *Arabidopsis* seedlings with and without inoculation of strain AD13−4 grew well, with no significance. Under alkaline–sodic conditions (pH 8.0 + 1.5 mM NaHCO_3_), although *Arabidopsis* seeds germinated, the roots did not elongate and the cotyledons did not expand, indicating severe inhibition of seedling development. However, after inoculation with the strain AD13−4, the growth of the seedlings was restored, and the development of the aerial parts and roots were similar to those under normal conditions (Figure 1A,B). These results indicated that the strain AD13−4 effectively alleviated the growth inhibition of *Arabidopsis* seedlings under alkaline–sodic stress.

To confirm the plant growth–promoting effects of strain AD13−4, we conducted soil culture experiments with maize, rice, and alfalfa (the optimal concentration of carbonate solution for maize is 80 mM; for rice, 50 mM; and for alfalfa, 40 mM, respectively). The results showed that under saline–alkali conditions, the fresh weight, dry weight, plant height, root length, and chlorophyll contents significantly decreased (Figure 1C,D; Supplementary Figure S1), indicating inhibition of alkaline–sodic stress on photosynthesis and plant biomass. The activities of antioxidant enzymes (superoxide dismutase, peroxidase, and ascorbate peroxidase) significantly increased (Figure 1F; Supplementary Figure S1F), and consistent with this, the ROS-scavenging capacities significantly increased (Figure 1G), indicating an increase in ROS levels which was induced by saline–alkali stress. The malondialdehyde (MDA) content also significantly increased (Figure 1E), indicating that the increased ROS level caused oxidative damage to the membranes. After inoculation of the strain AD13−4, the fresh weight, dry weight, plant height, and chlorophyll contents of the plants significantly increased (Figure 1D,E), indicating an enhancement of photosynthesis and plant growth and development. The antioxidant enzyme activities and ROS-scavenging capacities also significantly increased, while the content of MDA was significantly reduced (Figure 1F,G), suggesting that the strain AD13−4 improved plant antioxidant capacities, which alleviated oxidative damage to the membranes. The contents of total proteins and sugars significantly increased, while that of proline significantly decreased (Figure 1E), suggesting that plant metabolism was regulated by strain AD13−4 to adapt to saline–alkali condition. All these results indicate that strain AD13−4 regulates plant antioxidant capacities and metabolic processes to adapt to saline–alkali conditions and improves plant growth and development. And the growth-promoting effects of strain AD13−4 are efficient and broad–spectrum.

### 2.2. Identification and Characteristics of Strain AD13−4

**Figure 2:**
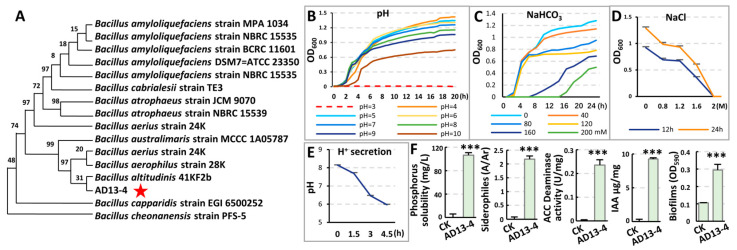
Identification and characteristics of strain AD13−4. (**A**) Phylogenetic tree of strain AD13−4. Genus of strain AD13−4 was identified by 16S rDNA sequencing. MEGA X was used to construct the phylogenetic tree. Neighbor–joining was used, bootstrap replications = 1000. (**B**–**D**) Detection of pH (**B**), alkali (NaHCO_3_, (**C**)), and salt (NaCl, (**D**)) tolerance of strain AD13−4. (**E**) Detection of H^+^ secretion ability of strain AD13−4. The pH value of LB medium (pH 8.1) rapidly decreased after inoculation of strain AD13−4. (**F**) Determination of strain AD13−4’s abilities to dissolve phosphorus; secrete siderophiles; ACC deaminase, and IAA; and produce biofilms. For the statistics, three biological replicates per sample were used. ***, *p* < 0.001. ns, no significance. Student’s *t*−test.

To identify the genus of strain AD13−4, the 16S rRNA fragment was amplified and sequenced. By blasting the NCBI database and phylogenetic tree analysis, strain AD13−4 was identified as a bacterium belonging to the genus *Bacillus altitudinis*, with 99.16% homology with the *Bacillus altitudinis* 41KF2b (Figure 2A). The AD13−4 colonies on nutrient agar were yellow and transparent, convex with a regular margin, and 2–3 mm in diameter after 14 h incubation. Strain AD13−4 utilized tartrate, Simmons’s citrate, and malonate as the sole carbon sources, respectively (Supplementary Table S2). In summary, strain AD13−4 is a novel bacterium belonging to the genus *Bacillus altitudinis*.

As a PGPR that functions under saline–alkali conditions, strong salt and alkali tolerance is necessary. First, we checked pH tolerance of strain AD13−4. The OD_600_ value of the bacterial culture with different pH showed that strain AD13−4 could not survive under the pH 3 condition, but could grow well under pH 4–9 conditions. Under the pH 10 condition, the concentration of bacteria culture after 20 h cultivation was significantly lower than those under pH 4–9 conditions, which was 52.5% of that under the pH 4 condition (Figure 2B). These results indicate that strain AD13−4 has a wide pH adaptation range.

Next, the alkali tolerance of strain AD13−4 was checked. The concentrations of bacterial culture after 24 h cultivation with NaHCO_3_ indicated no significant difference in the growth of strain AD13−4 under a 40 mM NaHCO_3_ condition. As the concentration of NaHCO_3_ increased, the proliferation ability of strain AD13−4 decreased, but it could still maintain a certain degree of growth and reproduction. Even when the NaHCO_3_ concentration reached 200 mM, strain AD13−4 could still survive (Figure 2C), indicating that strain AD13−4 has a strong tolerance to alkali stress. Then, the salt tolerance of strain AD13−4 was checked. The concentrations of bacterial culture showed that strain AD13−4 could grow and proliferate in a high NaCl concentration of 1.6 M, but could not survive when the NaCl concentration reached a high concentration of 2 M (Figure 2D), indicating that strain AD13−4 has a strong tolerance to salt stress.

To confirm the effects of secretions of strain AD13−4 on plants, *Arabidopsis* seeds were sown on the regular or carbonate medium containing cell−free fermentation broth of AD13−4. While, the seedlings didn’t show significance compared to those grown with inoculation of strain AD13−4. To investigate the growth−promoting mechanism of strain AD13−4, its secretions were detected. Firstly, the ability of strain AD13−4 to secrete acidic substances was tested. After inoculation of strain AD13−4 into LB medium with pH 8.1, the pH value rapidly decreased to 5.98 after 4.5 h cultivation (Figure 2E), indicating that strain AD13−4 has a strong ability to secrete acidic substances under alkali conditions, which could alleviate the alkalinity of the rhizosphere soil and facilitate root development. Further detection indicated that strain AD13−4 exhibits the activities of solubilizing phosphorus; fixing nitrogen; and producing siderophores, IAA, ACC deaminase, biofilms, and growth-promoting volatile substances (Figure 2F; Supplementary Figure S2A,B). These results indicate that strain AD13−4 can provide available phosphorus, iron ions, and nitrogen sources for plant growth; colonize on the root surface by forming biofilms; regulate plant auxin levels; and alleviate the inhibitory effects of ethylene on plant growth.

### 2.3. Strain AD13−4 Regulated Endogenous Phytohormone Levels and Cell Division Activity under Alkaline–Sodic Stress

**Figure 3:**
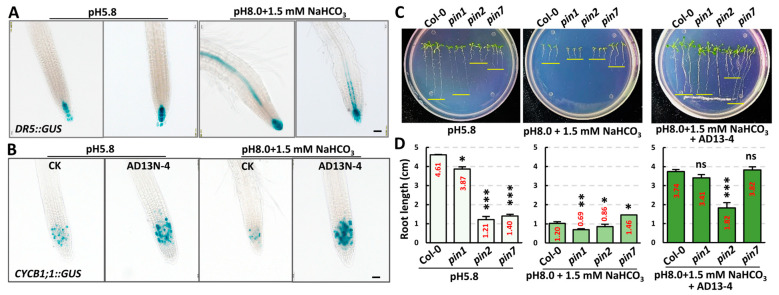
*Bacillus altitudinis* AD13−4 affected plant endogenous auxin level and cell division activity. The chemical staining of roots of 7−day−old *DR5::GUS* (**A**), *CYCB1;1::GUS* (**B**) expressing seedlings. Bars = 50 μm. (**C**) 12−day−old seedlings of the indicated genotypes. The yellow lines indicate tips of the roots. (**D**) Statistics of the root length of seedlings in (**C**). For the statistics, three independent experiments per sample were used. n = 30. *, *p* < 0.05; **, *p* < 0.01; ***, *p* < 0.001. ns, no significance. Student’s *t*-test. The number shown inside each column indicate the statistical value.

The phytohormone auxin plays a major role in the entire process of plant development and stress response. Auxin enhances plant development by regulating cell division, tissue expansion, stimulus response, etc. [46]. The PIN−FORMED (PIN) auxin exporters redirect auxin fluxes in response to environmental stimuli via their dynamic polar subcellular localizations [47]. Due to the alleviation of root development inhibition by strain AD13−4 under alkaline–sodic stress, we examined its effects on cell division activity using *CYCB1;1::GUS*, and on the auxin level using *DR5::GUS* reporter lines, respectively. *CYCB1;1* is required for cell division in the M phase, and its expression is significantly induced in response to auxin [48]. On the other hand, cytokinin and auxin jointly regulate plant development [49]. The histochemical staining results indicated that under alkaline–sodic stress, the *DR5::GUS* signals increased markedly in the stele and columella cells (Figure 3A), while *CYCB1;1::GUS* signals decreased dramatically in the root apical meristem (RAM) (Figure 3B), suggesting that excessive accumulation of the auxin inhibited cell division and root elongation. After inoculation of strain AD13−4, the auxin level decreased significantly and cell division activity was restored, suggesting that the strain AD13−4 can regulate endogenous phytohormone levels to promote cell division and root elongation. To verify the effects of the strain AD13−4 on plant endogenous hormone levels, we observed the growth phenotypes of the *pin* mutants after inoculating strain AD13−4 under saline–alkali conditions. As shown in Figure 3C,D, under normal conditions, the root lengths of *pin1*, *pin2* and *pin7* were significantly shorter than those of the wild type, especially *pin2* and *pin7*. Under alkaline–sodic conditions (pH 8.0 + 1.5 mM NaHCO_3_), the root lengths of Col-0, *pin1*, and *pin2* significantly decreased, while that of *pin*7 did not show significant change and was longer than that of Col-0. After inoculation with strain AD13−4, the root lengths of Col-0 and *pin* mutants significantly increased. There was no significant difference between Col-0, *pin1*, and *pin7*, while root length of *pin2* was significantly shorter than these lines. These results indicated that strain AD13−4 significantly affected auxin transport and distribution as well as cell division activity, thereby promoting plant development under saline–alkali stress. The different change patterns in the root length of the *pin* mutants after the treatments indicated that PIN1, PIN2, and PIN7 have different response mechanisms to alkaline–sodic stress and strain AD13−4.

The outermost layer of cells at the tip of the root cap that are about to detach or have already detached from the root cap are called border cells. The differentiation of border cells is regulated by stem cell activity and auxin [50]. Under alkaline–sodic conditions, the differentiation frequency of border cells with application of strain AD13−4 was significantly higher than that without AD13−4 (Supplementary Figure S2C,D). This result suggested that strain AD13−4 may modulate cell differentiation via affecting root stem cell activity and auxin levels.

### 2.4. Transcriptome Analysis of Alfalfa root Response to Strain AD13−4 under Alkaline–Sodic Conditions

To elucidate the molecular mechanisms of plant response to strain AD13−4 under alkaline–sodic stress, we conducted transcriptome analysis using alfalfa roots treated with a 40 mM carbonate solution. The experimental materials were divided into three groups: CK (treated with water), SAS (saline–alkali stress, treated with carbonate solution), and SAS + AD13−4. A total of 599.97 Mb of clean reads were obtained, with a Q30 > 93.5% and GC content ranging from 41.34% to 42.08%. These results confirmed the high quality of the assembled transcripts.

**Figure 4:**
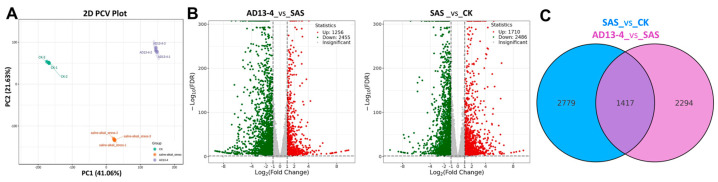
General overview of differentially expressed genes. (**A**) PCA diagram. Green, CK; orange, SAS; purple, SAS + AD13−4. *X*- and *Y*-axis indicate the first and second principal components (PC1 and PC2), respectively. The score plot of PC1 indicates cohesion within each group and that of PC2 indicates separation between the groups. (**B**) Volcano map of transcriptome genes for AD13−4_vs._SAS and SAS_vs._CK. Red dots, upregulated genes; green dots, downregulated genes; gray dots, genes with no significance. (**C**) Venn diagram of DEGs for AD13−4_vs._SAS and SAS_vs._CK.

The principal component analysis (PCA) revealed significant differences among the three groups (Figure 4A). The PC1 score plots indicated good cohesion within each group, and the PC2 score plots indicated significant separation between the groups. A total of 6490 differentially expressed genes (DEGs) were identified using DESeq2 R package (v. 1.22.1). In the SAS_vs._CK group, there were 1256 DEGs upregulated and 2455 downregulated, while in AD13−4_vs._SAS group, there were 1710 DEGs upregulated and 2,486 downregulated DEGs (Figure 4B). Venn-diagram analysis indicated that there were 2779 and 2294 DEGs unique to the SAS_vs._CK and AD13−4_vs._SAS groups, respectively, while 1417 DEGs were common to the two groups (Figure 4C).

**Figure 5:**
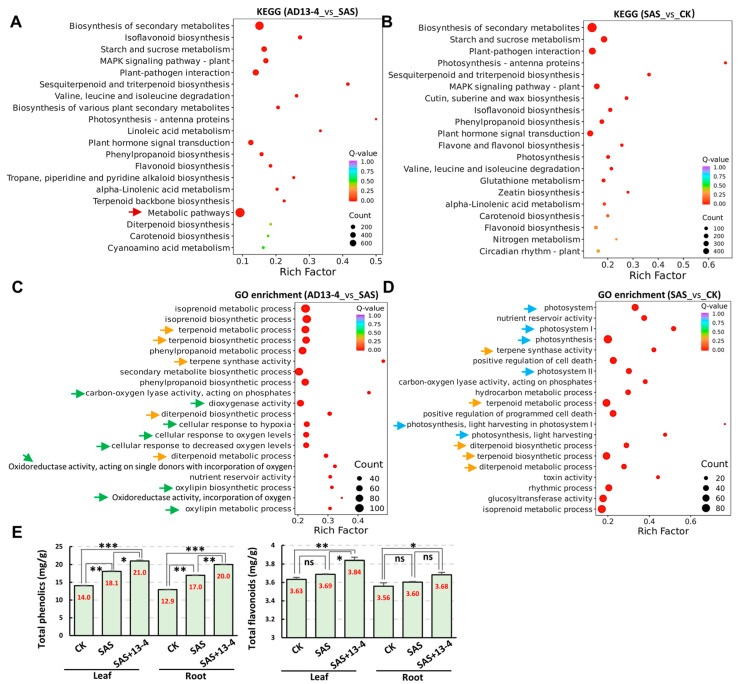
Comparative transcriptome analysis of DEGs. KEGG analysis of AD13−4_vs._SAS (**A**) and SAS_vs._CK (**B**) and GO enrichment analysis of AD13−4_vs._SAS (**C**) and SAS_vs._CK (**D**) indicated significant changes under alkaline–sodic stress and application of strain AD13−4. The color of the circle represents the *Q* value, and the size of the circle represents the gene number. Orange arrows, terpenoid biosynthesis pathways; green arrows, redox reaction pathways; blue arrows, photosynthesis pathways. (**E**) Statistics of contents of total phenolics and total flavonoids. For the statistics, three biological replicates per sample. *, *p* < 0.05; **, *p* < 0.01; ***, *p* < 0.001. ns, no significance. Student’s *t*-test. The numbers shown inside each column indicate the statistics value.

The comparison of Kyoto Encyclopedia of Genes and Genomes (KEGG) pathways between AD13−4_vs._SAS and SAS_vs._CK groups showed significant changes in many pathways under both AD13−4 and SAS conditions, including secondary metabolism, photosynthesis, signal transduction, plant–pathogen interactions, phenylpropanoid biosynthesis, etc. (Figure 5A,B). In addition, in the AD13−4_vs._SAS group, there were significant changes in metabolic pathways (Figure 5A, arrow). These results indicated that the above pathways responsive to alkaline–sodic stress were also regulated by strain AD13−4, thereby alleviating the impacts of alkaline–sodic stress on alfalfa.

Gene ontology (GO) analysis showed that the DEGs were classified into thirty-two subcategories in three main categories: fifteen subcategories in Biological Processes (BP), two in Cellular Components (CC), and fifteen in Molecular Functions (MF). The DEG distribution patterns in AD13−4_vs._SAS and SAS_vs._CK groups were similar (Supplementary Figure S3). A comparison of GO enrichment pathways between AD13−4_vs._SAS and SAS_vs._CK indicated significant enrichment in the terpenoid biosynthesis and metabolic pathways in both groups (Figure 5C,D, orange arrows), while the redox reaction pathways were specifically enriched in AD13−4_vs._SAS (Figure 5C, green arrows) and the photosynthesis pathways were specifically enriched in SAS_vs._CK (Figure 5D, blue arrows), indicating that these processes were important for plants to adapt to the alkaline–sodic environments, or for strain AD13−4 to alleviate alkaline–sodic stress. We also detected the contents of total phenols and total flavonoids. The results indicated that under alkaline–sodic stress, the total phenolic content significantly increased, but the total flavonoid content had no significance. After inoculation of strain AD13−4, both of them significantly increased (Figure 5E), indicating that strain AD13−4 can regulate secondary metabolic processes, thereby improving plant alkaline–sodic tolerance.

Due to the significant difference in DEG enrichment between AD13−4_vs._SAS and SAS_vs._CK, we analyzed the expression of the common DEGs shown in the Venn plot. The results indicated that 114 of the common genes were downregulated under alkaline–sodic stress and upregulated after inoculation of AD13−4. Conversely, 327 of the common genes were upregulated under alkaline–sodic stress and downregulated after inoculation of strain AD13−4 (Supplementary Figure S4), suggesting that these DEGs were not only specifically regulated by alkaline–sodic stress, but also by strain AD13−4 to improve plant adaptation to alkaline–sodic stress.

### 2.5. Analysis of Signaling Pathways Responsive to Strain AD13−4 under Alkaline–Sodic Stress

**Figure 6:**
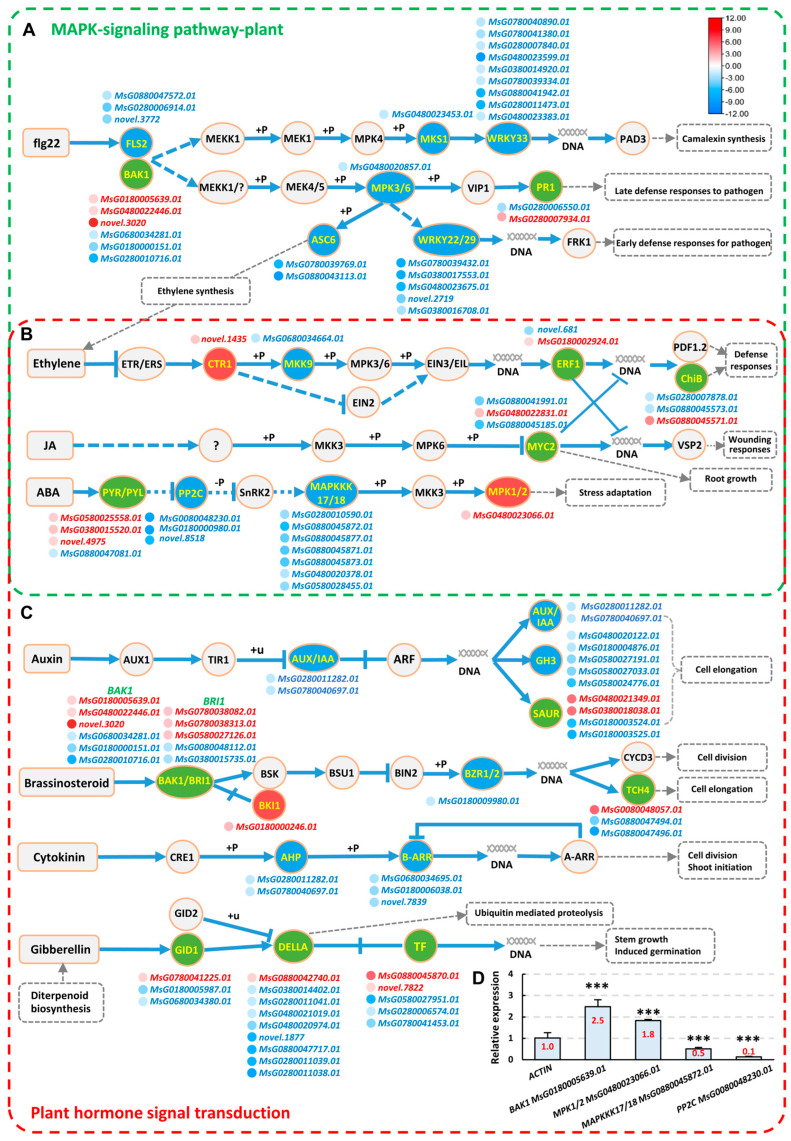
The signal transduction pathways in AD13−4_vs._SAS group. (**A**–**C**) MAPK signaling pathways (green frame) and Plant hormone signal transduction pathways (red frame), summarized according to Supplementary Figure S5. The overlapped parts are highlighted in (**B**). Rectangles, pathways; red circles, upregulated genes; blue circles, downregulated genes; green circles, gene family containing both upregulated and downregulated genes. The dots indicate genes. The colors of the dots indicate significances shown in the color scale. Gray dotted lines and rectangles indicate downstream pathways. (**D**) Statistics of relative expression of genes (part) in the signaling pathways. Three independent experiments per sample, three replicates per experiment. ***, *p* < 0.001. Student’s *t*-test. The number shown inside each column indicate the statistical value. Abbreviations: A−AAR, *Arabidopsis* type−A activators response regulators; ABA, abscisic acid; ARF, auxin response factor; AHP, histidine−containing phosphotransmitters; B−AAR, *Arabidopsis* type−B activators response regulators; BAK1, BRI1−associated kinase1; BIN2, BR−insensitive2; BRI1, Brassinosteroid–insensitive1; BSK, Brassinosteroid signaling kinase; BSU1, BR−suppressor1; BZR1, brassinazole resistant1; ChiB, Bacterial chitinase; CRE1, cytokinin response1; CTR1, copper uptake protein1; CYCD3, *Arabidopsis* cyclin D3; ETR1, Ethylene Receptor1; ERS, ethylene response sensor; EIN3/EIL, Ethylene−insensitive3; ERF1, ethylene response factor1; flg22, Flagellin22; FLS2, flagellin receptor2; FRK1, Fructokinase1; GH3, glycoside hydrolase3; GID2, GA−insensitive Dwarf2; IAA, Indole−3−acetic acid; JA, Jasmonic acid; MEKK1, mitogen−activated protein kinase kinase kinase1; MEK1, mitogen−activated protein kinase1; MPK4, mitogen−activated protein kinase4; MKS1, map kinase substrate1; MYC, myelocytomatosis; PAD3, Peptidylarginine deiminase3; PP2C, protein phosphatases 2C; PR1, pathogenesis−related protein1; PYR, pyrabactin resistance; PYL, pyrabactin resistance−like; SnRK2, sucrose non−fermenting related kinase Group2; TCH4, *Arabidopsis* plants harboring4; TF, transcription factor; TIR, Toll−like, interleukin−1 receptor, resistance protein; SAUR, small auxin−up RNA; VIP1, VirE2−interacting protein1; VSP2, vegetative storage protein2.

We conducted an in−depth analysis of the signaling pathways of the AD13−4_vs._SAS group. The signal transduction pathways of alfalfa in response to AD13−4 under alkaline–sodic conditions include two pathways, the MAPK signaling pathway and plant hormone signal transduction. The MAPK signaling pathways included pathogen infection (flg22), pathogen attack (H_2_O_2_), phytohormone (JA, ethylene, and ABA), and ROS–related pathways (Figure 6, green frame, part of the pathways; Supplementary Figure S5A). And the plant hormone signal transduction pathways included auxin, cytokinin, gibberellin, ABA, ethylene, JA, SA, and Brassinosteroid–related pathways (Figure 6, red frame, part of the pathways; Supplementary Figure S5B). These two processes had some overlapping parts, such as the ethylene, JA, and SA−related pathways (Figure 6B). Moreover, the flg22−induced pathways were also included in plant–pathogen interaction pathways (Figure 6A). The expression levels of many key genes in these pathways were altered significantly after inoculation of AD13−4, such as *FLS2*, *BAK1*, *MPK1*/*2*, *PR1*, *MYC2*, *SAUR*, *GELLA*, *PP2C*, etc., indicating that strain AD13−4 activated the signaling pathways to enable plants to respond and adapt to alkaline–sodic stress. The RT−qPCR validation results of some of the genes were consistent with those of the transcriptome (Figure 6D). The above results indicate that the strain AD13−4 regulates plant tolerance to alkaline–sodic stress by affecting signal transduction pathways.

In the stress response signaling pathways, transcription factors (TFs) serve as bridges, transmitting stimulus signals by binding to cis–regulatory elements in the promoters of the target genes. A total of 243 differentially expressed TFs were identified. The top five families were AR2/ERF, MYB, NAC, WRKY, and bHLH, which broadly respond to both biotic and abiotic stresses (Supplementary Figure S6). Interestingly, compared to the seven bZIP and eight C2C2–Dof TFs identified in SAS_vs._CK group, only one bZIP and four C2C2–Dof TFs were identified in AD13−4_vs._SAS group. Moreover, the TF number in MYB and bHLH families in SAS_vs._CK group significantly decreased compared to those in SAS_vs._CK group (Supplementary Figure S6B). The bZIP, MYB, and bHLH family TFs are frequently involved in plant stress responses, and the Dof family is also reported to be involved in saline–alkali stress response in rice [1]. The decrease in the number of these TFs in AD13−4_vs._SAS group reflected that the strain AD13−4 alleviated the impacts of alkaline–sodic stress on plants.

### 2.6. Analysis of Metabolic Pathways Responded to Alkaline–Sodic Stress

**Figure 7:**
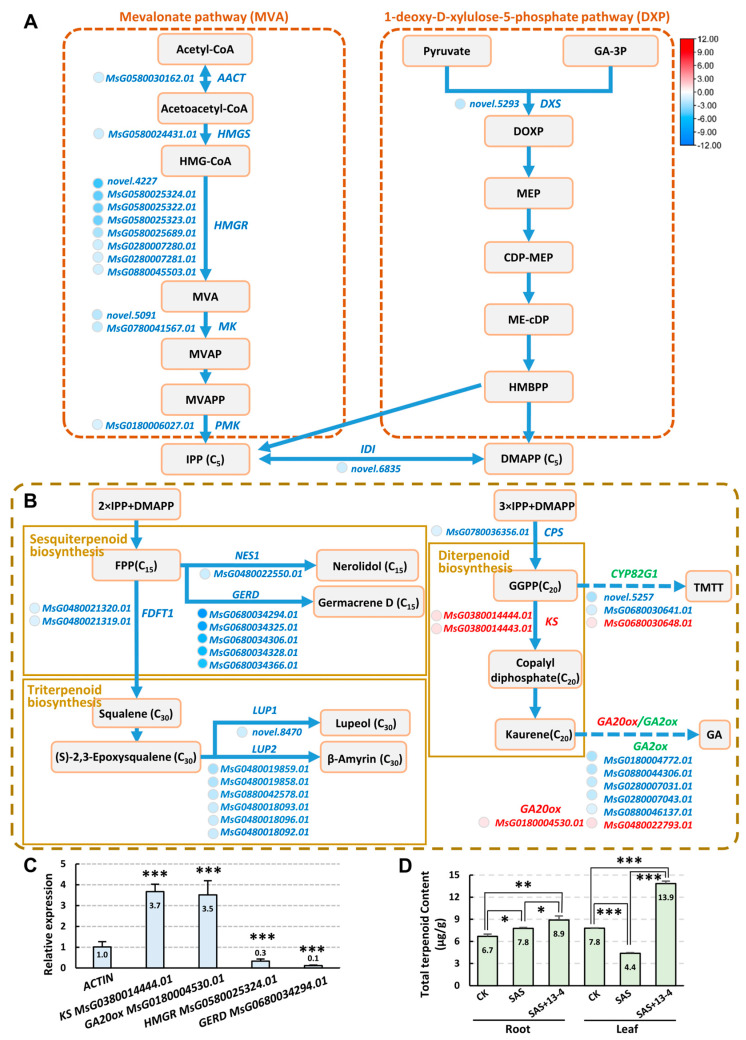
The terpenoid biosynthesis pathways in AD13−4_vs._SAS group. (**A**) MVA and MEP pathways which generate IPP and DMAPP, respectively. (**B**) The sesquiterpenoid, triterpenoid, and diterpenoid biosynthesis pathways. (**C**) Statistics of relative expression of some of the genes determined by RT−qPCR. Three independent experiments per sample, three replicates per experiment. (**D**) Statistics of content of total terpenoids. The standard curve is y = 0.0108x + 0.0214, R^2^ = 0.9995. Three biological replicates per sample. *, *p* < 0.05; **, *p* < 0.01; ***, *p* < 0.001. Student’s *t*-test. The numbers shown inside each column indicate the statistical value. Abbreviations: CPS, Copalyl di-phosphate synthase; CDP, 2−C−methyl−D−erythritol 2,4−cyclodiphosphate synthase; CDP−ME, 24−(cytidine 5′−diphospho)−2−C−methyl−d−erythritol; DOXP, 1−deoxy−D−xylulose−5−phosphate; DMBPP, 3−(2−Hydroxyphenyl)−3,3−dimethylpropanoate; FDFT1, farnesyl−diphosphate farnesyltransferase; FPP, farnesyl diphosphate; GA−3P, glyceratdehyde−3−phosphate, GGPP, geranylgeranyl diphosphate; HMBPP, (E)−4−hydroxy−3−methyl−but−2−enyl diphosphate; HMG−CoA, 3−hydroxy−3−methylglutaryl−CoA; HMGR, 3−hydroxy−3−methylglutaryl−CoA reductase; IPP, Isopentenyl diphosphate; MVA, mevalonate; MVAP, mevalonate 5−phosphate, MVAPP, 5−pyrophosphomevalonate; MEP, methylerythritol 4−phosphate. NES, nerolidol synthase; TMTT, 4,8,12−trimethyltrideca−1,3,7,11−tetraene.

In plants, all terpenoids are derived from isopentenyl diphosphate (IPP) and its enzymatically interconvertible isomer dimethylallyl diphosphate (DMAPP), which are generated from the mevalonate (MVA) and 2−C−methyl−D−erythritol−4−phosphate (MEP/DXP) pathways, respectively [51] (Figure 7A). In the MVA pathway, most of the synthetase genes were downregulated, suggesting a possible reduction in IPP contents. In addition, downregulation of *IDI*, the IPP–DMAPP converting enzyme gene, may also lead to a decrease in IPP and DMAPP production. In sesquiterpenoid, triterpenoid, and diterpenoid biosynthesis pathways, the expression levels of many synthase genes had significant changes (Figure 7B). The RT−qPCR results of some of the genes were consistent with those of the transcriptome (Figure 7C), suggesting potential changes in the production of terpenoids. To verify the speculation, we determined the contents of total terpenoids. Under alkaline–sodic conditions, the total terpenoids significantly increased in the roots, but sharply decreased in the leaves, while after inoculation of strain AD13−4, the total terpenoids significantly increased in both the roots and leaves, especially in the leaves, and the total terpenoid contents increased by over three times compared to that before inoculation (Figure 7D). These results indicate that the strain AD13−4 regulated the plant secondary metabolism to enable plants to adapt to alkaline–sodic soil.

### 2.7. Impacts of Strain AD13−4 on Rhizosphere Bacterial Community

**Figure 8:**
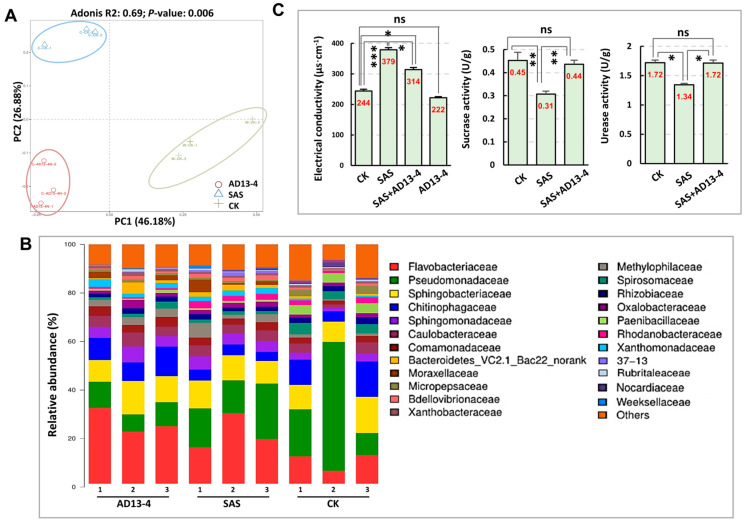
The impacts of AD13−4 on the abundance and composition of rhizosphere microbiota. (**A**) PCA diagram. Red circle, AD13−4; blue circle, SAS; green circle, control (SAS + AD13−4). *X*- and *Y*-axis indicate the first and second principal components (PC1 and PC2), respectively. Score plots of PC1 and PC2 show cohesion within each group and separation between the groups, respectively. (**B**) Analysis of relative abundance of dominant families. (**C**) Statistics of soil activity indicators, electrical conductivity, and activities of sucrase and urease. For the statistics, three biological replicates per sample were used. *, *p* < 0.05; **, *p* < 0.01; ***, *p* < 0.001. ns, no significance. Student’s *t*-test. The number shown inside each column indicates the statistical value.

Since PGPR can promote soil metabolism, which is mainly related to the soil microbial community [52], we conducted 16S rRNA gene sequencing of the alfalfa rhizosphere microbiome. The scatter diagrams of PCA with the first (PC1) and the second component (PC2) indicated that the cumulative contribution rates of CK, SAS, and AD13−4 groups were 46.18%, 26.88%, and 63.56%, respectively (Figure 8A). The PC1 score plots indicated the stability and repeatability of the results within each group, and the PC2 score plots indicated a significant difference between the groups (Figure 8A). Analysis of the 16S rRNA gene sequencing results yielded a total of 584,753 optimized sequences and 246,817,741 bases. The similarity of the operational taxonomic unit (OTU) was 97% with a classification confidence of 70% in the optimized reads (with a length of ≥ 400 bp), and the coverage rate of all samples was above 97%, indicating that the sequencing results were reliable. The inoculation of strain AD13–4 did not significantly affect the alpha diversity of bacteria in alkaline–sodic soil (Supplementary Figure S7A). The dominant bacterial phyla were *Bacteroidota*, *Proteobacteria*, *Firmicutes*, *Bdellovibrionota*, and *Verrucomicrobiota*. And the community barplot analysis indicated a significant alteration in the composition and proportion of bacterial families in the rhizosphere soil (Figure 8B). The most abundant bacteria were *Flavobacteraceae*, *Pseudomonadaceae*, *Sphingobacteriaceae*, and *Chitinophagaceae*. After application of strain AD13−4, the proportions of *Flavobacteraceae*, *Chitinophagaceae*, *Caulobacteraceae*, etc., increased; while those of *Pseudomonadaceae*, *Rhodanobacteraceae*, *Bdellovibrioneceae*, etc., decreased; and 37–13 and *Weeksellaceae* almost disappeared (Figure 8B). These results indicated that strain AD13−4 had impacts on abundance and composition of rhizosphere microbiota in alkaline–sodic soil.

The cladogram, which explains the evolutionary relationships and biodiversity between species, indicated that the rhizosphere microbiota among AD13−4, SAS, and CK groups had significant differences (Supplementary Figure S7B). Lefse analysis of biomarkers showed that *Feruginibacter*, *Polaromonas*, and *Nubsella* were the biomarkers in strain AD13−4−treated alkaline–sodic soil rather than in alkaline–sodic soil and CK soil (Supplementary Figure S7C), suggesting that these bacteria were recruited to the alfalfa rhizosphere by strain AD13−4.

The activity of soil enzymes, a key indicator of soil fertility and abiotic stress, is significantly affected by the soil microbial community [52]. The determination of alfalfa rhizosphere soil properties showed that the electrical conductivity increased significantly, and the activities of urease and sucrase were seriously inhibited under alkaline–sodic stress. However, after inoculation of strain AD13−4, the electrical conductivity significantly decreased, and the activities of urease and sucrase were restored (Figure 8C), indicating that strain AD13−4 can improve the properties and activities of alkaline–sodic soil.

## 3. Discussion

The various secretions of strain AD13−4 affect the levels of endogenous phytohormones, which in turn affect plant metabolism; the cell division activity of the meristem; as well as cell differentiation, e.g., root cap and border cell. The root border cells protect continuously growing root tips by secreting compounds such as proteins, polysaccharides, phytoalexins, mucus, organic acids, etc. [50,53]. The border cells are crucial for root growth and plant health, as they contribute to nutrient accumulation around the roots and may cause changes in the rhizosphere microbial population by improving the physical and chemical properties of rhizosphere soil and recruiting beneficial microorganisms, thereby affecting the diversity, abundance and composition of rhizosphere microbiota. The metabolites secreted by border cells also have defensive functions [54]. For example, a certain flavonoid compound in alfalfa roots can inhibit the growth of trichomonas in soil [55]. The detachment frequency of border cells is regulated by a complicated mechanism involving the expression of a series of TFs, quiescent center identity, stem cell activity, and auxin distribution and concentration [50]. In our study, the frequency of boundary cell generation was significantly higher in the present of strain AD13−4 (Supplementary Figure S2C,D), suggesting that AD13−4 may accelerate border cell differentiation by affecting endogenous phytohormone levels. In turn, increased abundance of rhizospheric microbiota rewards vigorous plant growth and stress tolerance, indicating a mutually beneficial interaction between plants and PGPRs [35,55,56]. The increase in the abundance of beneficial microorganisms in rhizosphere soil promotes carbon and nitrogen cycling in soil, which helps to improve the effectiveness of nutrients in the soil [57].

The application of strain AD13−4 affected plant physiological processes, e.g., primary and secondary metabolism. In the secondary metabolic processes, the terpenoid and flavonoid biosynthesis pathways were altered significantly after the application of strain AD13−4. Terpenoids are the most abundant and structurally diverse metabolites in plants, and play a crucial role in plant growth and development and adaptation to environments [56,58,59,60,61]. In plants, all terpenoids are derived from IPP and DMAPP, which are generated from the MVA and MEP/DXP pathways, respectively [51]. The total terpenoid contents in alfalfa roots and leaves showed different change patterns under alkaline–sodic stress (Figure 7D). This phenomenon suggested a different response of roots and leaves to alkaline–sodic stress, or/and a long−distance transport of terpenoids from leaves to roots directly struggling with alkaline–sodic stress. After the application of strain AD13−4, the total terpenoid contents increased significantly in both roots and leaves (Figure 7D), indicating that strain AD13−4 activated the terpenoid biosynthesis pathways in both tissues to improve plant tolerance to stress.

In the MVA pathway, 3–hydroxy–3–methylglutaryl–CoA reductase (HMGR) is the first rate–limiting enzyme, closely related to oxidative stress, proliferation, ER morphogenesis, and plant response to hormones [51]. IPP is not only a precursor of terpenes, but is also used to produce brassinosteroids, cytokinins, and phytosterols, which are crucial for cell membrane fluidity and plant growth and development [62,63]. After the application of strain AD13−4, the *HMGR* expression levels altered significantly, which may have affected IPP contents and, subsequently, antioxidant capacities, endogenous phytohormone levels, cell activities, etc., in plants. Geranylgeranyl diphosphate (GGPP) is a substrate not only for the synthesis of diterpenoids, but also for several important plant hormones, such as gibberellin, abscisic acid, and strigolactone. In addition, GGPP is a precursor of carotenoids and chlorophyll and an important joint in several important secondary metabolic pathways in plants [58]. The changes in upstream and downstream gene expression levels (Figure 7B,C) may cause changes in GGPP contents, thereby affecting phytohormone levels and photosynthesis. All the changes suggest crucial roles of the strain AD13−4 in the regulation of plant growth and development and adaptation to environmental stresses.

There was also a significant change in phenylpropanoid biosynthesis in KEGG enrichment analyses (Figure 5A,B). Phenylpropanoid metabolites mainly include flavonoid and lignin biosynthesis pathways. Salt stress induces the biosynthesis of flavonoid compounds, which in turn act as antioxidants to reduce salt stress–induced oxidative damage [6]. Studies on tomato roots found a significant increase in phenylpropane synthesis genes and metabolites under salt stress [49]. It was recently reported that, to adapt to high saline–alkali stress, rice leaves accumulate a large amount of lipids, organic acids, organic oxygen compounds, phenylpropanoids, and polyketides [1]. In alfalfa roots, a large amount of DEGs were enriched in Phenylpropanoid biosynthesis pathways after application of strain AD13−4, and the increase in total flavonoid contents once again emphasized the importance of this pathway in plant adaptation to alkaline–sodic environment.

The inoculation of *Bacillus altitudinis* AD13−4 significantly improved the physical and chemical properties and activities of soil, as well as rhizospheric microbiota composition and abundance, which might contribute to the alleviation of alkaline–sodic stress on plants. At the family level, the relative abundance of *Flavobacteraceae* and *Chitinophagaceae*, which belong to *Bacteroidota*, increased after inoculation of AD13−4. It has been reported that the bacteria in *Bacteroidota* might enhance soluble phosphorus in soil by secreting phosphorus−solubilizing enzymes. And *Bacteroidota* and *Firmicutes* can synergistically degrade rice straw in paddy fields [64]. Furthermore, the relative abundance of some biomarkers, *Caulobacteraceae*, *Feruginibacter*, *Polaromonas*, and *Nubsella,* increased after applying strain AD13−4. Many strains of *Caulobacteraceae,* which belongs to *Proteobacteria,* have the ability to fix nitrogen and participate in the nitrification process to increase soil nutrients [29,65]. *Feruginibacter* is often found in activated sludge for treating various types of wastewater, secrets a large amount of extracellular polymers, and is related to the formation of sludge flocs and biofilms [66,67,68,69]. *Polaromonas* has been reported to potentially influence plant acclimation and resilience to cold stress [70]. And *Nubsella zeaxanthinifaciens* gen. nov., sp. nov. belongs to the family *Sphingobacteriaceae* and produces Zeaxanthin, the major carotenoid pigment [71]. The carotenoids act as a source of retrograde signals with impacts on plant development and stress responses [72]. *Flavobacteriaceae* is considered as a key polysaccharide-degrading bacterium [73]. The organic macromolecules in nature can be hydrolyzed by microorganisms, e.g., *Flavobacteriaceae* strain F89T [19], and used as a source of nutrition and energy, which are beneficial for plant growth and adaptation to the environment. *Flavobacterium* strain TRM1 can suppress *R. solanacearum* disease development in a susceptible plant, revealing its role in protecting plants from microbial pathogens [74]. The beneficial bacteria (which could be called PGPR) promote nitrogen, carbon, and phosphorus cycling by increasing soil enzyme activities to increase soil nutrient contents and enhance plant resistance to biotic and abiotic stress. The increase of above bacteria suggest that strain AD13−4 plays a crucial role in recruiting beneficial bacteria to enhance soil activity, plant growth and development, as well as disease resistance. Biofilms facilitate the colonization of PGPRs on the surfaces of roots, thereby achieving a stable growth–promoting function [51]. The strong ability of strain AD13−4 to form biofilms suggests its high affinity to plant roots, thus providing conditions for better functional utilization.

Our reevaluation of the molecular mechanism of alfalfa root response to *Bacillus altitudinis* AD13−4 provided potential genetic targets for developing alkaline–sodic-tolerant plants. On the other hand, strain AD13−4 can survive well in alkaline–sodic soil, revealing its capacity to colonize and adapt to the interface between soil and plants, which provides a theoretical basis for its application to the improvement of alkaline–sodic land.

## 4. Materials and Methods

### 4.1. Plant Materials and Treatments

*Arabidopsis* seeds (Col-0, *DR5::GUS*, *CYCB1;1::GUS*, *and pin1/2/7*) were surface−sterilized with 75% (*v*/*v*) ethanol for 5 min and then washed three times with sterile H_2_O. The sterilized seeds were sown on regular medium (1/2MS, pH 5.8) or carbonate medium (1/2MS, pH 8.0 + 1.5 mM NaHCO_3_), which simulated alkaline–sodic conditions. Vertical cultivation was utilized at 22 °C, with 16 h light/8 h dark. To investigate the effects of the secretions of strain AD13−4 on plants, rather than the bacterium itself, *Arabidopsis* seedlings were photographed before their root tips came into contact with the bacterium. To further confirm the effects of the secretions of strain AD13−4 on plants, its cell−free fermentation broth was used for the germination of *Arabidopsis* seeds. The bacterium was cultured overnight in 1/2MS liquid medium, and the concentration was adjusted to OD_600_ = 1, then centrifuged. The supernatant was filtered by a 0.22 μm filter, and then 200 μL of it was applied to the surface of 1/2MS medium or carbonate medium and air−dried. Then, the seeds were sown on the medium with/without the cell−free fermentation broth and vertically cultivated.

For the soil culture, the carbonate solution containing Na_2_CO_3_:NaHCO_3_ = 1:9 (molar ratio) was used to simulate the carbonate composition in Soda saline–alkali land in Northeast China. The treatment was applied once every seven days, a total of three times. Rice (*Oryza sativa*) seeds germinated at 28 °C for five days were sown in peat soil (PINDSTRUP SUBSTRATE, 5−20 mm, 330 L), with a total of 30 seedlings (three pots per treatment and 10 seedlings per pot). The control group (CK) was treated with water, the SAS group was treated with 50 mM carbonate solution, and the SAS + AD13−4 group was treated with carbonate solution and strain AD13−4. The growth conditions were 26 °C and 10 h light/14 h dark, with a humidity level of 50–70%. Maize seeds (B73, a commercial cultivar in Heilongjiang Province) were sown in peat soil, with a total of 15 seeds (three pots per treatment and 5 seeds per pot). The seeds were treated with water (CK), 80 mM carbonate solution (SAS), or carbonate solution and AD13−4 (SAS + AD13−4). The growth conditions were 22 °C and 16 h light/8 h dark, with a humidity level of 50–70%. Alfalfa (*Medicago sativa* L.) seeds were sown in peat soil, with a total of 180 seeds (three pots per treatment and 60 seeds per pot). On the fifth day after germination, the seedlings were treated with water (CK), 40 mM carbonate solution (SAS), and carbonate solution and strain AD13−4 (SAS + AD13−4). The growth conditions were 22 °C and 16 h light/8 h dark, with a humidity level of 50−70%. For alkaline–sodic treatment, the rate of carbonate solution:soil (*v*/*v*) was about 2:5. Strain AD13−4 (OD_600_ = 1) was inoculated to the soil [1:50 (*v*/*v*)] during the first treatment. For the first treatment, a double volume of carbonate solution was used to thoroughly irrigate the soil.

### 4.2. Screening of PGPR and Molecular Identification of Strain AD13−4

For isolation of the rhizosphere microorganism, five grams of rhizosphere soil from native plants grown in Soda saline–alkali land in Northeast China (Anda, Heilongjiang Province, China) was added to 45 mL of sterile water, stirred for 15 min, and stewed for 10 min, then 1 mL of the supernatant was taken and added to 9 mL of sterile water and mixed well (the dilution was recorded as 10^−1^). Then, 1 mL of it was taken, added to 9 mL of sterile water, and mixed well (the dilution was recorded as 10^−2^), and so on to prepare bacterial suspensions with different dilutions of 10^−3^, 10^−4^, 10^−5^, 10^−6^, and 10^−7^. A sample of 0.1 mL of each dilution was evenly spread on LB solid medium and incubated at 30 °C for 2–3 d. Single colonies were selected to separate the bacteria by streaking more than 3 times to obtain a single strain.

To screen PGPR, 1/2MS medium (pH 8.0) containing 1.5 mM NaHCO_3_ was used to simulate the alkaline–sodic condition, and *Arabidopsis* Col-0 was used as the plant material. Strains that could significantly promote the growth of *Arabidopsis* seedlings were considered to be PGPR, including AD13−4. The 16S rRNA gene of AD13−4 was PCR−amplified with specific primers (listed in Supplementary Table S3), and the PCR products were determined by Sanger sequencing. The 16S rRNA sequence was uploaded (gene bank number OR863787, NCBI number SUB14003048) (Supplementary Figure S8). Via blasting in NCBI databases (rRNA/ITS databases), AD13−4 was identified as a bacterium belonging to the genus *Bacillus altitudinis*. MEGA X was used to construct the phylogenetic tree. Neighbor−joining was used, with 1000 bootstrap replications. The physiological and biochemical identification of *Bacillus altitudinis* AD13−4 was conducted using Micro−biochemical identification tubes (HOPEBIO, Shanghai, China).

### 4.3. Physiological Determination and Histochemistry Assay

A quantity of 0.2 g roots of one−month−old alfalfa plants were collected for physiological measurement. Briefly, guaiacol colorimetry was used for the determination of the POD activity, nitro blue tetrazolium photoreduction for SOD activity, and the hydrogen peroxide method for CAT activity, while APX activity was determined according to the reduction in the AsA content [75]. The MDA content was determined using the thiobarbituric acid method, and the proline content using the acidic ninhydrin colorimetry method [1,75]. Three biological replicates per sample were used. Total antioxidant capacity assay kits (Shanghai Enzyme-linked Biotechnology Co., Ltd., Shanghai, China) were used to gauge the radical scavenging capacities of DPPH (515 nm), ABTS (734 nm), and FRAP (593 nm). Three biological replicates per sample were used [76,77]. For the GUS (β–glucuronidase) staining assay, the seedlings were incubated in a GUS staining buffer [80 mM sodium phosphate buffer (pH 7.0), 0.4 mM potassium ferricyanide, 0.4 mM potassium ferrocyanide, 8 mM EDTA, 0.05% Triton X−100, 0.8 mg/mL 5−bromo−4−chloro−3−indolyl−β−D−glucuronide] for 4 h at 37 °C [78]. Three independent experiments per sample were used.

### 4.4. Determination of Total Phenols, Total Flavonoids, and Total Terpenoids

The root material was the same as that used for physiological determination in 4.3. The procedures for the determination of total flavonoids and total phenolics referred to [75,78]. For determination of total terpenoids, 10 mL of methanol was added to 0.1 g of powder of the tissues, ultrasonicated (80 kW) for 30 min, and left stewing overnight at 4 °C; then, the supernatant was used as a test solution. For standard solution preparation, 25, 50, 100, 150, 200, 300, and 400 μL of 1 mg/mL ursolic acid solution were evaporated dry in an 80 °C water bath, then 200 μL of 5% vanillin ice acetic acid solution and 400 μL of concentrated sulfuric acid were added and incubated in a 60 °C water bath for 15 min, and 5 mL of glacial acetic acid was added. The same volumes of standard solutions were added in the proper order to the test solutions. The absorbance value was determined at 543 nm. The test solutions without addition of ursolic acid were used as control. Three biological replicates per sample were used [79].

### 4.5. Determination of Salt, Alkali, and pH Tolerance of Strain AD13−4

To investigate the pH tolerance range of strain AD13−4, 1 mL of AD13−4 culture (OD_600_ = 1) was added to 100 mL of LB liquid medium with different pH values (pH 3−10) and cultured at 30 °C for 20 h. The concentration of the bacterial culture was measured every 2 h and the growth curve was plotted. To investigate the alkali tolerance of strain AD13−4, 1 mL of AD13−4 culture (OD_600_ = 1) was added to 100 mL LB liquid medium with different concentrations of NaHCO_3_ (0−200 mM) and cultured at 30 °C for 24 h. The concentration of the bacterial culture was measured every 2 h, and the growth curve was plotted. To investigate the salt tolerance of strain AD13−4, 1 mL of AD13−4 culture (OD_600_ = 1) was added to 100 mL LB liquid medium with different concentrations of NaCl (0−2 M) and cultured at 30 °C for 24 h. The concentration of the bacterial culture was measured at 12 h and 24 h, and the growth curve was plotted. To determine the H^+^ secretion ability, 1 mL of AD13−4 culture (OD_600_ = 1) was added to 100 mL LB liquid medium (pH 8.1) and cultured at 30 °C for 4.5 h. The pH value was measured every 1.5 h.

### 4.6. Determination of Characteristics of Strain AD13−4

To detect the nitrogen fixation capacity of strain AD13−4, Ashby’s Mannitol Agar medium (10 g/L mannitol, 0.2 g/L KH_2_PO_4_, 0.2 g/L MgSO_4_·7H_2_O, 0.2 g/L NaCl, 0.1 g/L CaSO_4_·2H_2_O, 5 g/L CaCO_3_) was used. To detect the ability to produce ACC deaminase, DF and ADF medium (T10407, T10408, Saint–bio, Shanghai, China) were used. To detect the ability to produce IAA, the Salkowski colorimetric reaction was conducted. To detect the ability to produce siderophiles, MKB liquid medium (casamino acid 5.0 g, glycerol 15 mL, K_2_HPO_4_ 2.5 g, MgSO_4_·7H_2_O 2.5 g, H_2_O 1000 mL, pH 7.2) was used. To detect the phosphate solubilization ability, the Molybdenum antimony colorimetric method was employed. To detect the ability to generate biofilms, the Giemsa staining method was used.

### 4.7. Detection of Rhizospheric Soil Enzymatic Activities

After removing loose soil from the roots, a thin layer of soil attached to the alfalfa root surface was collected as rhizosphere soil. The 3,5−dinitrosalicylate colorimetric method was used to determine sucrase activity at 508 nm. Sodium phenolate was used to determine urease activity at 578 nm [80].

The electrical conductivity (EC) of the saturated soil extract (soil:water = 1:5, *w*/*v*) was determined using an EC meter (DDS–11A, Shang Hai Yoke Instrument Co., Ltd., Shanghai, China). The pH values were measured using the saturated soil extract with a pH meter [80]. The rhizosphere soil was the same as that used for 16S rRNA gene sequencing.

### 4.8. Alfalfa RNA Isolation, Library Construction, RNA Sequencing, and RT−qPCR

The roots used for RNA sequencing were the same as those used for physiological determination in 4.3. The total RNA was extracted using the TRIzol reagent (Invitrogen, Carlsbad, CA, USA) according to the manufacturer’s protocol. The procedures of library construction, RNA sequencing, and RT−qPCR referred to [1]. For RT−qPCR, three independent experiments per sample and three replicates per experiment were used. The primer sequences are listed in Supplementary Table S3. Raw transcriptome reads were deposited in the NCBI Sequence Read Archive (SRA) database (PRJNA1025112).

### 4.9. Preparation and 16S rRNA Gene Sequencing of Rhizospheric Microbiota

The rhizosphere soils of one−month−old alfalfa plants were collected. The procedure of 16S rRNA gene sequencing referred to [80]. Three replicates per sample were used. Briefly, microbial genomic DNA was extracted using the TruSeqTM DNA Sample Prep Kit (Illumina, San Diego, CA, USA), and the hypervariable region (V3–V4) of the bacterial 16S rRNA genes was amplified using the primers 338F and 806R in an GeneAmp^®^ 9700 PCR thermocycler (Applied Biosystems, CA, USA). The PCR products were purified using an AxyPrep DNA Gel Extraction Kit (Axygen Biosciences, Union City, CA, USA) and quantified using a Quantus™ Fluorometer (Promega, Beijing, China). Purified amplicons were pooled in equimolar amounts and paired−end−sequenced on an Illumina MiSeq PE300 platform (Illumina, San Diego, CA, USA) according to the standard protocols of Majorbio Bio−Pharm Technology Co., Ltd. (Shanghai, China). Raw reads were deposited in the NCBI Sequence Read Archive (SRA) database (PRJNA1028144). The phylogenetic tree was constructed by selecting the *Bacillus* strains in NCBI database with the highest identity of 16S rRNA sequences to those of AD13−4. MEGA X was used to construct the phylogenetic tree.

### 4.10. Bioinformatics Analysis

For transcriptomic analysis, alfalfa RNA was extracted according to the protocol using CTAB-PBIOZOL reagent. After identifying and quantifying total RNA using a bioanalyzer (Thermo Fisher Scientific, MA, USA), the mRNA was purified using Oligo(dT) magnetic beads. The cDNA was generated by a reverse transcription kit and then amplified by PCR. The products were purified using Agencourt Ampure XP Beads (A63882, BECKMAN), and then dissolved with EB solution. The double-stranded PCR products were denatured and cycled using a cleaver oligonucleotide sequence. The single-stranded circular DNA (ssCir DNA) format was used for the final library. The library was sequenced on the Illumina HiSeq X Ten platform, and the raw data were filtered using fastp (v 0.23.2), mainly to remove reads with adapters. HISAT was used to locate the clean reads to the alfalfa genome (‘Zhongmu No.1’ alfalfa). FPKM was calculated based on the length of each gene and the number of reads mapped to the genes. The DESeq2 R package (v. 1.22.1, |log_2_foldchang| ≥ 1 and FDR < 0.05) [81,82] was used to analyze the differential expression between each two groups, and the *p* value was corrected using the Benjamini and Hochberg method [1]. The enrichment analysis was performed based on the hypergeometric test. For KEGG, the hypergeometric distribution test was performed with the unit of the pathway; for GO, it was performed based on the GO term [1,83].

For rhizospheric microbiota analysis, paired–end reads from the original DNA fragments were merged using FLASH (v 1.2.11, default). Paired-end reads were assigned to each sample according to the unique barcodes. Sequence analysis was performed using the UPARSE software package (v 7.0.1090, http://drive5.com/uparse/, accessed on 20 March 2024) with the UPARSE–OTU and UPARSE–OTUref algorithms. Sequences with ≥97% similarity were assigned to the same OTUs. Representative sequences were picked for OTUs, and the RDP classifier was used to annotate taxonomic information for each representative sequence. In order to compute alpha diversity, we rarified the OTU table and calculated three metrics: Chao1 estimated the species abundance; Observed Species estimated the amount of unique OTUs found in each sample; and the Shannon index was calculated. Cluster analysis was preceded by principal component analysis (PCA), which was applied to reduce the dimensions of the original variables using the QIIME software package (v 1.9.0, default). QIIME was used to calculate both the weighted and unweighted unifrac distance, which are phylogenetic measures of beta diversity. Unweighted unifrac distance was used for principal coordinate analysis (PCoA) and the unweighted pair group method with arithmetic mean (UPGMA) clustering. To confirm differences in the abundances of individual taxonomy between the two groups, Metastats software (v 1.0, default) was utilized. LEfSe was used for the quantitative analysis of biomarkers within different groups to provide biological class explanations to establish statistical significance, biological consistency, and effect–size estimation of the predicted biomarkers [80].

## 5. Conclusions

Our results show that *Bacillus altitudinis* AD13−4 can significantly improve plant tolerance to alkaline–sodic stress by improving antioxidant capacities, endogenous phytohormone levels, cell division activity, and cell differentiation. Transcriptome analyses indicate that metabolism/secondary metabolism, signaling, photosynthesis, redox reaction, and plant–pathogen interaction pathways were significantly altered under alkaline–sodic stress and the application of strain AD13−4. Consistent with this, the contents of many metabolites and secondary metabolites, e.g., proline, phenolics, flavonoids, and terpenes, which are crucial for plant development and adaptation to the environment, changed significantly. Our results, to some extent, elucidate the mechanism underlying the interaction between *Bacillus altitudinis* AD13−4 and alfalfa roots under alkaline–sodic stress and provide clues for improving bacterial strains for biofertilizer.

## Data Availability

All data are presented in this article.

## Supplementary Materials

The following supporting information can be downloaded at: https://www.mdpi.com/article/10.3390/ijms25115785/s1.
