# Supplementary material for: Bacillus altitudinis AD13−4 Enhances Saline–Alkali Stress Tolerance of Alfalfa and Affects Composition of Rhizosphere Soil Microbial Community
Source: Int J Mol Sci. 2024 May 26;25(11):5785. doi: 10.3390/ijms25115785 (PMC11171787; doi:10.3390/ijms25115785)
Supplement: Supplementary file 1 [file ijms-25-05785-s001.zip › AD13-4 Supplementary fiures.pdf]

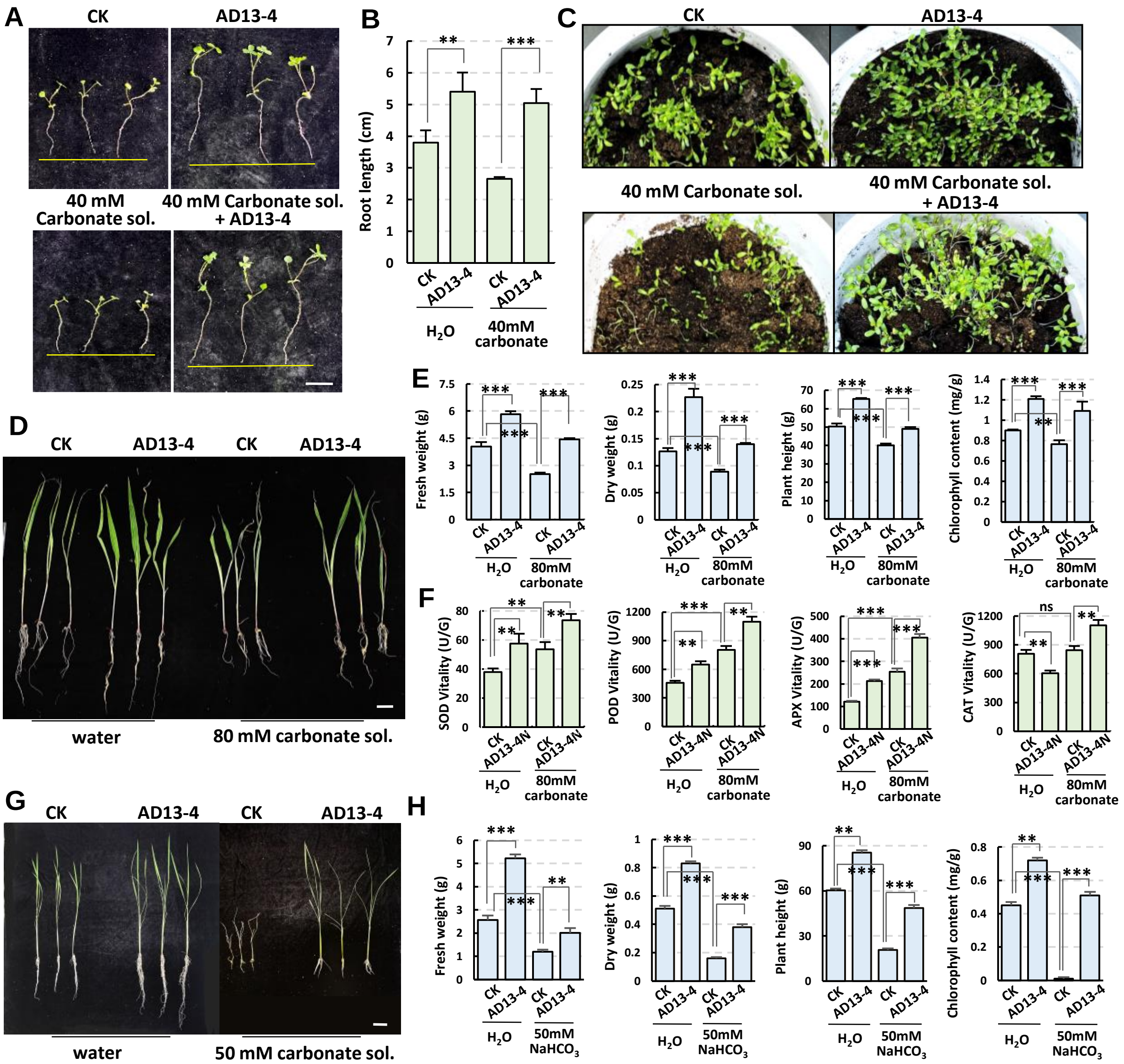

Supplementary Figure S1 *Bacillus altitudinis* AD13-4 promoted plant growth with broad-spectrum and high efficiency. **(A, B)** Phenotypes of alfalfa seedlings **(A)** and statistics of root length **(B)**. The lines indicate tips of the roots. Bars=2 cm. **(C)** The growing status of alfalfa seedlings in soil. **(D, G)** Phenotypes of seedlings of maize **(D)** and rice **(G)** under saline-alkali stress. **(E, F and H)** Statistics of the physiological indexes of maize **(E, F)** and rice **(H)**. Bars = 5 cm. For the statistics, three independent experiments per sample. Student's t test. *, $P < 0.05$; **, $P < 0.01$; ***, $P < 0.001$. CK, blank; ns, no significance; SAS, saline-alkali stress; sol., solution.

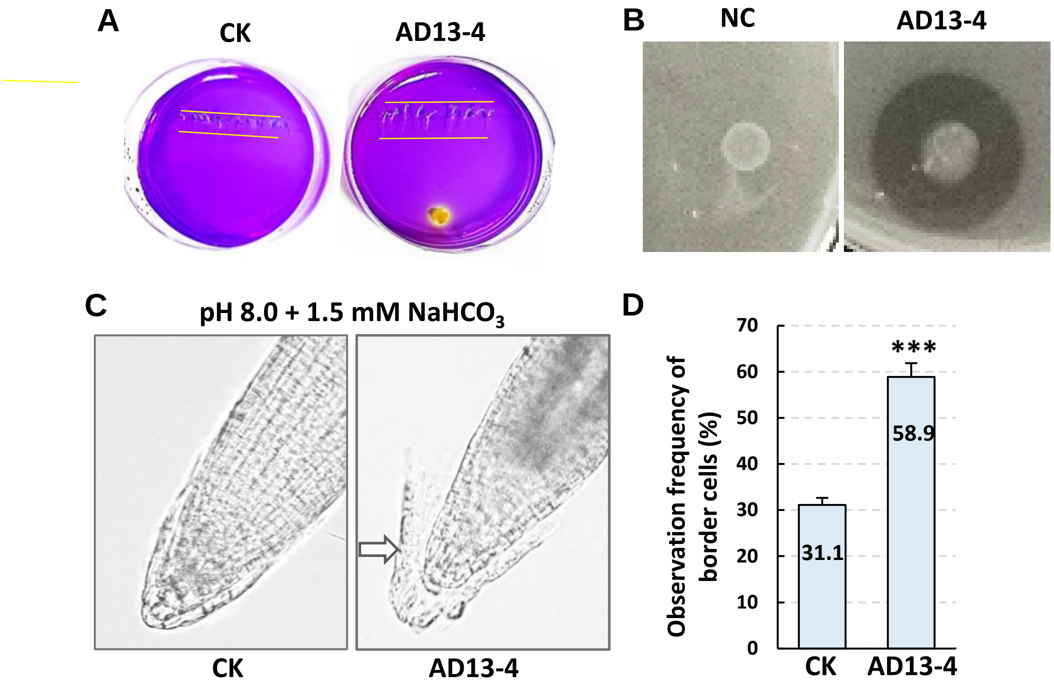

Supplementary Figure S2 Detection of secretions of *Bacillus altitudinis* AD13-4. **(A)** Detection of volatile substances of strain AD13-4. *Arabidopsis* seeds were sown on 1/2MS medium (pH6.8) containing 0.003% (w/v) bromocresol violet, a pH indicator, with (right panel)/without (left panel) inoculation of strain AD13-4, cultivated for 7 d. The bromocresol violet changed to yellow under acidic condition. The lines indicate tips of the shoots and roots. **(B)** Detection of the nitrogen fixation ability of AD13-4.**(C)** Root border cells (arrow) observed under saline-alkali condition. **(D)** Statistics of observation frequency of root border cells. Three independent experiments per sample. ***, $P < 0.001$. Student's t test. The number shown inside each column indicates the statistics value. CK, blank; NC, negative control.

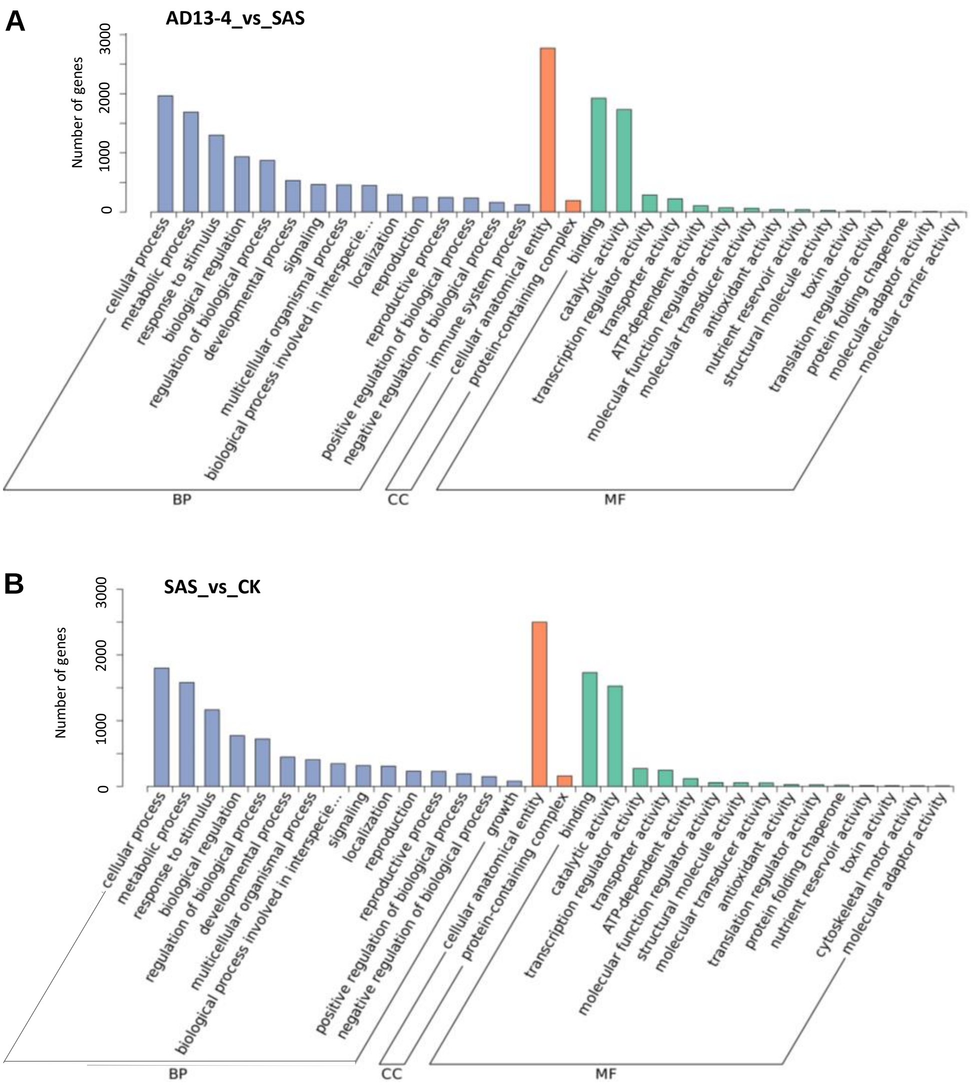

Supplementary Figure S3 GO categorization of AD13-4 vs SAS **(A)** and SAS vs CK **(B)**. BP, biological process; MF, molecular function; CC, cellular component. The Y axis represents the gene number. The blue columns represent BP, the orange columns represent CC, and green columns represent MF.

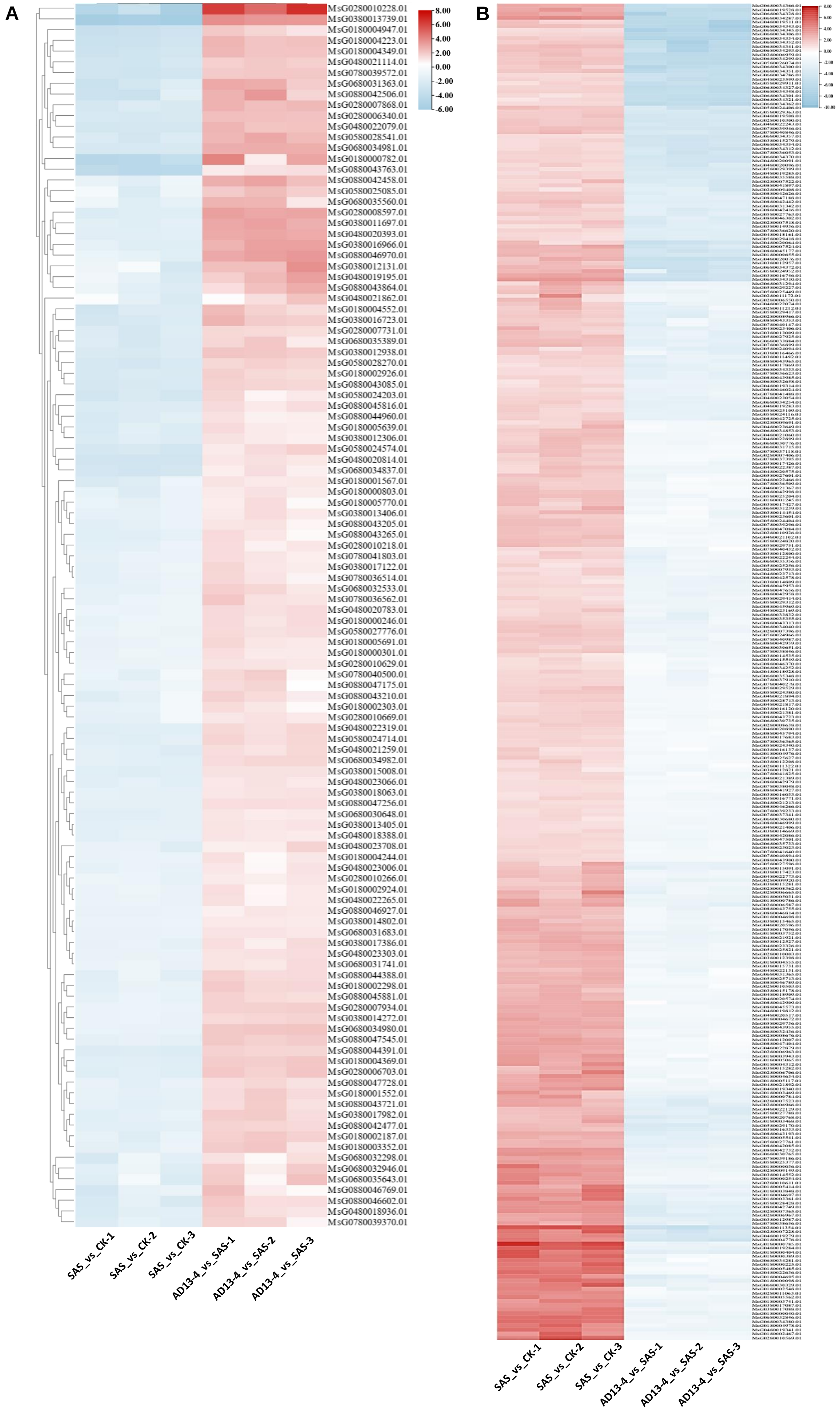

Supplementary Figure S4 Different expression patterns of the common DEGs in SAS vs CK and AD13-4 vs SAS groups shown in the Venn plot. **(A, B)** Upregulated **(A)** and downregulated **(B)** DEGs after applying AD13-4

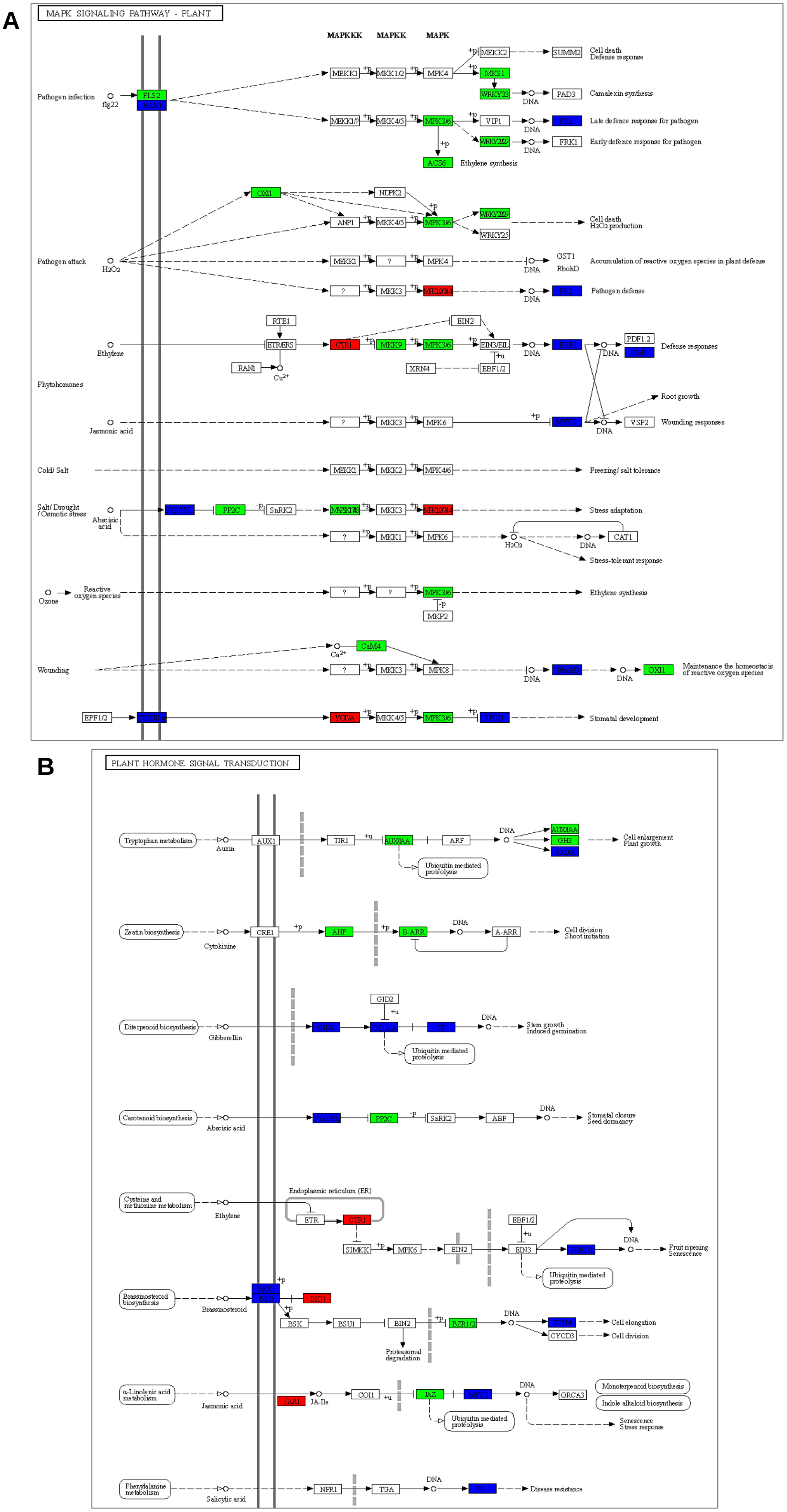

Supplementary Figure S5 The signal transduction pathways. **(A)** MAPK signaling pathway. **(B)** Plant hormone signal transduction.

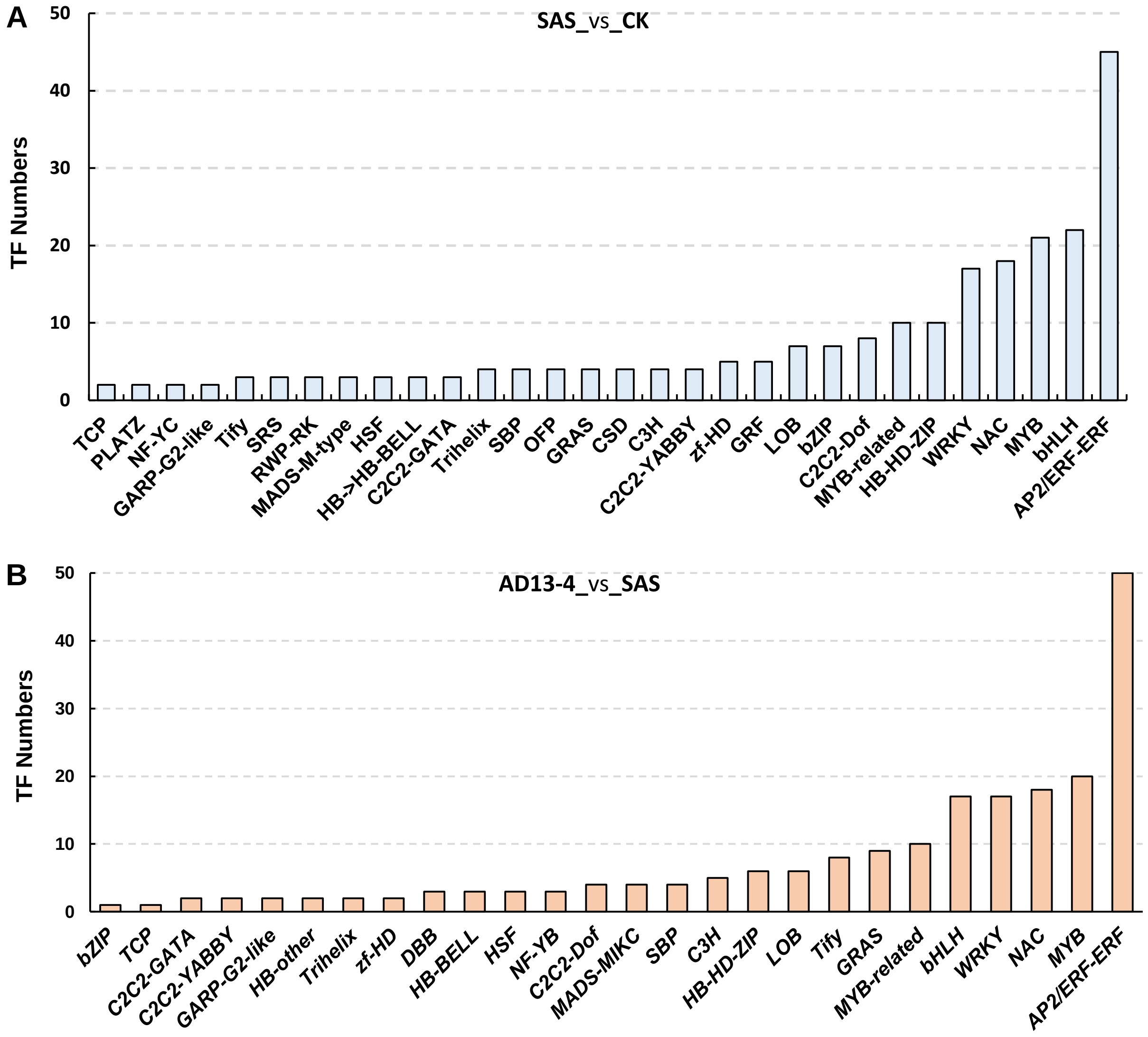

Supplementary Figure S6 Distribution of differentially expressed transcription factors. **(A)** TF families in SAS vs CK group. **(B)** TF families in AD13-4 vs SAS group. The clades in X axis indicate TF families, and Y axis represents the TF number.

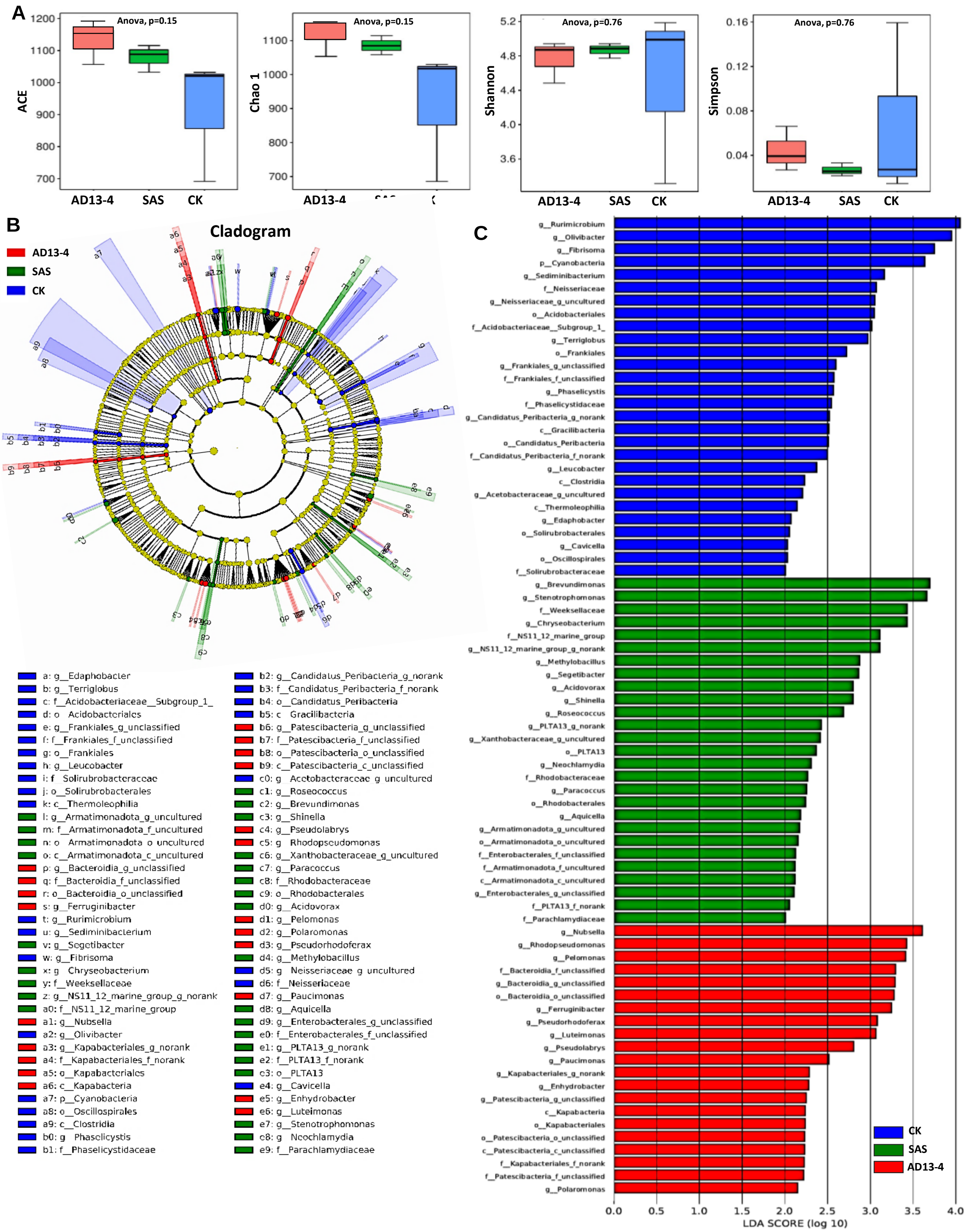

Supplementary Figure S7 The alpha diversity and LEfSe analysis of rhizosphere microbiota. **(A)** The alpha diversity analysis of rhizospheric microbiota. **(B, C)** Analysis of Cladogram **(B)** and LDA score **(C)**.

TATACTGCAGTCGAGCGGACAGAAGGGAGCTTGCTCCCGGATGTTAGCGGCGG
ACGGGTGAGTAACACGTGGGTAACCTGCCTGTAAGACTGGGATAACTCCGGGA
AACCGGAGCTAATACCGGATAGTTCCTTGAACCGCATGGTTCAAGGATGAAAGA
CGGTTTCGGCTGTCACTTACAGATGGACCCGCGGCGCATTAGCTAGTTGGTGAG
GTAACGGCTCACCAAGGCGACGATGCGTAGCCGACCTGAGAGGGTGATCGGCC
ACACTGGGACTGAGACACGGCCCAGACTCCTACGGGAGGCAGCAGTAGGGAA
TCTTCCGCAATGGACGAAAGTCTGACGGAGCAACGCCGCGTGAGTGATGAAGG
TTTTCGGATCGTAAAGCTCTGTTGTTAGGGAAGAACAAGTGCAAGAGTAACTGC
TTGCACCTTGACGGTACCTAACCAGAAAGCCACGGCTAACTACGTGCCAGCAGC
CGCGGTAATACGTAGGTGGCAAAGGCGTTGTCCGGAATTATTGGGCGTAAAG
GGCTCGGAGGCGGTTTCTTAAGTCTGATGTGAAAGCCCCCGGCTCAACCGGG
GAGGGTCATTGGAAACTGGGAAACTTGAGTCCAGAAGAGGAGAGTGGAATTC
CCACGTGTAGCGGTGAAATGCGTAGAGATGTGGAGGAACACCCAGTGGCGAAG
GCGACTCTCTGGTCTGTAACTGACGCTGAGGAGCGAAAGCGTGGGGAGCGACC
AAGGATTAGATACCCTGGTAGTCCACGCCGTAAACGATGAGTGCTAAGTGTTAG
GGGGTTTCCGCCCCCTTAGTGCTGCAGCTAACGCATTAAGCACTCCGCCTGGGG
AGTACGGTCGCAAGACTGAAACTCAAAGGAATTGACGGGGGCCCGCACAAGC
GGTGGAGCATGTGGTTTAATTCGAAGCAACGCGAAGAACCTTACCAGGTCTTG
ACATCCTCTGACAACCCTAGAGATAGGGCTTTCCCTTCGGGGACAGAGTGACAG
GTGGTGCATGGTTGTCGTCAGCTCGTGTCGTGAGATGTTGGGTTAAGTCCCGCA
ACGAGCGCAACCCTTGATCTTAGTTGCCAGCATTCAGTTGGGCACTCTAAGGTG
ACTGCCGGTGACAAACCGGAGGAAGGTGGGGATGACGTCAAATCATCATGCCC
CTTATGACCTGGGCTACACACGTGCTACAATGGACAGAACAAAGGGCTGCGAG
ACCGCAAGGTTTAGCCAATCCCACAAATCTGTTCTCAGTTCGGATCGCAGTCTGC
AACTCGACTGCGTGAAGCTGGAATCGCTAGTAATCGCGGATCAGCATGCCGCGG
TGAATACGTTCCCGGGCCTTGTACACACCGCCCGTCACACCACGAGAGTTTGCA
ACACCCGAAGTCGGTGAGGTAACCTTTATGAGCCACCGC

Supplementary Figure S8 16S rDNA sequence of *Bacillus altitudinis* AD13-4.
