# Supplementary material for: Bacillus altitudinis AD13−4 Enhances Saline–Alkali Stress Tolerance of Alfalfa and Affects Composition of Rhizosphere Soil Microbial Community
Source: Int J Mol Sci. 2024 May 26;25(11):5785. doi: 10.3390/ijms25115785 (PMC11171787; doi:10.3390/ijms25115785)
Supplement: Supplementary file 1 [file ijms-25-05785-s001.zip › Supplementary Table S1.pdf]

Supplementary Table S1. Summary of the PGPRs mentioned in this study.

Bacterial Strain	Plant species	Products of PGPR	Stress type	References
Peribacillus frigoritolerans A70 *and Bacillus licheniformis* A46	Alfalfa (*Medicago sativa L.*)	ACC deaminase, IAA, siderophore and phosphate	Saline-alkaline	4
Bacillus subtilis (NBRI 28B), *B. subtilis* (NBRI 33 N), and *B. safensis* (NBRI 12 M)	Maiz (*Zea mays*)	ACC deaminase	Salinity	5
Enterobacter sp. PR14	Rice (*Oryza sativa L.)*	ACC deaminase, IAA, antioxidant enzymes and phosphate solublization	Salinity	21
Sinorhizobium meliloti GL1 *and Enterobacter ludwigii MJM-11*	Alfalfa (*Medicago sativa L.*)	Nodulation and nitrogen fixation	Saline-alkaline	23
Enterobacter asburiae D2	Rice (*Oryza sativ*a)	IAA, siderophore, ACC deaminase and phosphate solubilization	Salinity	24
Bacillus subtilis	Cotton (*Gossypium hirsutum*)	Nitrate and nitrogen fixation	Saline-alkaline	25
Pseudomonas aeruginosa and *Enterobacter aerogenes*	Alfalfa (*Medicago sativa L.*)	ACC deaminase, IAA, siderophore and phosphate	Saline-alkaline	26
Enterobacter bugandensis TJ6	Wheat (*Triticum aestivum L.*)	IAA, betaine, and arginine	Cd and Pb	27
Lysinibacillus fusiformis Cr33	Tomato	JA, IAA and NO	Salinity	28
Arthrobacter and *B. megaterium*	Tomato	IAA, phosphate solubilization	Saline-alkaline and salt stress	41
Acinetobacter sp.	oat (*A. sativa*)	ACC deaminase, IAA and phosphate solubilization	Saline–alkaline	43
Enterobacter and *Klebsiella*	Soybean	IAA, SA, taurine and Nitrogen fixation	Nitrogen	83
