# Supplementary material for: Bacillus altitudinis AD13−4 Enhances Saline–Alkali Stress Tolerance of Alfalfa and Affects Composition of Rhizosphere Soil Microbial Community
Source: Int J Mol Sci. 2024 May 26;25(11):5785. doi: 10.3390/ijms25115785 (PMC11171787; doi:10.3390/ijms25115785)
Supplement: Supplementary file 1 [file ijms-25-05785-s001.zip › Supplementary Table S2.pdf]

Supplementary Table S2. Chemotaxonomic characteristics of *Bacillus* sp.AD13-4. AD13-4 strain utilizes Tartrate, Simon citrate, and Malonate as the sole carbon source, but does not utilize Glucose fermentation, Lactose fermentation, Sucrose, Fructose, Mannitol, L-Rhamnose, Galactose, Acetate, Arabinose, and Glycerol. +, Positive; -, negative.

**Characteristic**	**AD13-4**
Tartrate	+
Glucose fermentation	-
Lactose fermentation	-
Simon citrate	+
Sucrose	-
Fructose	-
Malonate	+
Mannitol	-
L-Rhamnose	-
Galactose	-
Acetate	-
Arabinose	-
Glycerol	-
