# Supplementary material for: Bacillus altitudinis AD13−4 Enhances Saline–Alkali Stress Tolerance of Alfalfa and Affects Composition of Rhizosphere Soil Microbial Community
Source: Int J Mol Sci. 2024 May 26;25(11):5785. doi: 10.3390/ijms25115785 (PMC11171787; doi:10.3390/ijms25115785)
Supplement: Supplementary file 1 [file ijms-25-05785-s001.zip › Supplementary Table S3.pdf]

Supplementary Table S3. Primer sequence used in this study.

Gene ID	Primer sequence 5'-3'	Experiment
27F	AGAGTTTGATCCTGGCTCAG	16S rDNA amplification
1492R	GGTTACCTTGTTACGACTT	16S rDNA amplification
MsG0080048230.01_1_F	GGATTGTGGCGGAGGAGG	RT-qPCR
MsG0080048230.01_1_R	ATCACCAGCCACCTCCCT	RT-qPCR
MsG0180004530.01_1_F	CAGGACCACATTGTGACCCA	RT-qPCR
MsG0180004530.01_1_R	CGAGGTCTAACCGTTTGCCA	RT-qPCR
MsG0180005639.01_1_F	GTGATGGAGCAGAGCCGG	RT-qPCR
MsG0180005639.01_1_R	ACCTCCACCATTCACCGC	RT-qPCR
MsG0880045872.01_1_F	TTGGGTGTGCAGTGGTGG	RT-qPCR
MsG0880045872.01_1_R	GGCGATTCTTCTCCGGCA	RT-qPCR
MsG0580025324.01_1_F	CAACGGGCGATGCAATGG	RT-qPCR
MsG0580025324.01_1_R	CACTGCCGCGCTTTCTTG	RT-qPCR
MsG0480023066.01_1_F	CTGACAGGTGGCAGTGGG	RT-qPCR
MsG0480023066.01_1_R	GTCCGTTGCAGCAGGAGT	RT-qPCR
MsG0680034294.01_1_F	ACCCAGTGTTGGCTACGAC	RT-qPCR
MsG0680034294.01_1_R	GCTCAAACTCGCTTGACACA	RT-qPCR
MsG0380014444.01_1_F	TGCAAGGCAGAAACAAGCA	RT-qPCR
MsG0380014444.01_1_R	TGCCCAAGCTAAGCGAGA	RT-qPCR
338F	ACTCCTACGGGAGGCAGCAG	16S rRNA gene sequencing
806R	GGACTACHVGGGTWTCTAAT	16S rRNA gene sequencing
